# Unbalancing cAMP and Ras/MAPK pathways as a therapeutic strategy for cutaneous neurofibromas

**DOI:** 10.1172/jci.insight.168826

**Published:** 2024-01-04

**Authors:** Helena Mazuelas, Miriam Magallón-Lorenz, Itziar Uriarte-Arrazola, Alejandro Negro, Inma Rosas, Ignacio Blanco, Elisabeth Castellanos, Conxi Lázaro, Bernat Gel, Meritxell Carrió, Eduard Serra

**Affiliations:** 1 Hereditary Cancer Group, Translational Cancer Research Program, and; 2 Clinical Genomics Research Group, Germans Trias i Pujol Research Institute (IGTP), Can Ruti Campus, Badalona, Barcelona, Spain.; 3 Genetics Service, Germans Trias i Pujol University Hospital, Can Ruti Campus, Badalona, Barcelona, Spain.; 4 Hereditary Cancer Program, Catalan Institute of Oncology (ICO-IDIBELL), L’Hospitalet de Llobregat, Barcelona, Spain.; 5 Centro de Investigación Biomédica en Red de Cáncer, Spain.; 6 Departament de Fonaments Clínics, Facultat de Medicina i Ciències de la Salut, University of Barcelona, Barcelona, Spain.

**Keywords:** Cell Biology, Genetics, Cancer, Genetic diseases, Tumor suppressors

## Abstract

Cutaneous neurofibromas (cNFs) are benign Schwann cell (SC) tumors arising from subepidermal glia. Individuals with neurofibromatosis type 1 (NF1) may develop thousands of cNFs, which greatly affect their quality of life. cNF growth is driven by the proliferation of *NF1*^–/–^ SCs and their interaction with the *NF1*^+/–^ microenvironment. We analyzed the crosstalk between human cNF-derived SCs and fibroblasts (FBs), identifying an expression signature specific to the SC-FB interaction. We validated the secretion of proteins involved in immune cell migration, suggesting a role of SC-FB crosstalk in immune cell recruitment. The signature also captured components of developmental signaling pathways, including the cAMP elevator G protein–coupled receptor 68 (*GPR68*). Activation of Gpr68 by ogerin in combination with the MEK inhibitor (MEKi) selumetinib reduced viability and induced differentiation and death of human cNF-derived primary SCs, a result corroborated using an induced pluripotent stem cell–derived 3D neurofibromasphere model. Similar results were obtained using other Gpr68 activators or cAMP analogs/adenylyl cyclase activators in combination with selumetinib. Interestingly, whereas primary SC cultures restarted their proliferation after treatment with selumetinib alone was stopped, the combination of ogerin-selumetinib elicited a permanent halt on SC expansion that persisted after drug removal. These results indicate that unbalancing the Ras and cAMP pathways by combining MEKi and cAMP elevators could be used as a potential treatment for cNFs.

## Abstract 2

<p>A combination of MEK inhibition and cAMP elevation has potential for treating cutaneous neurofibromas by unbalancing RAS and cAMPsignaling pathways.</p>

## Graphical abstract

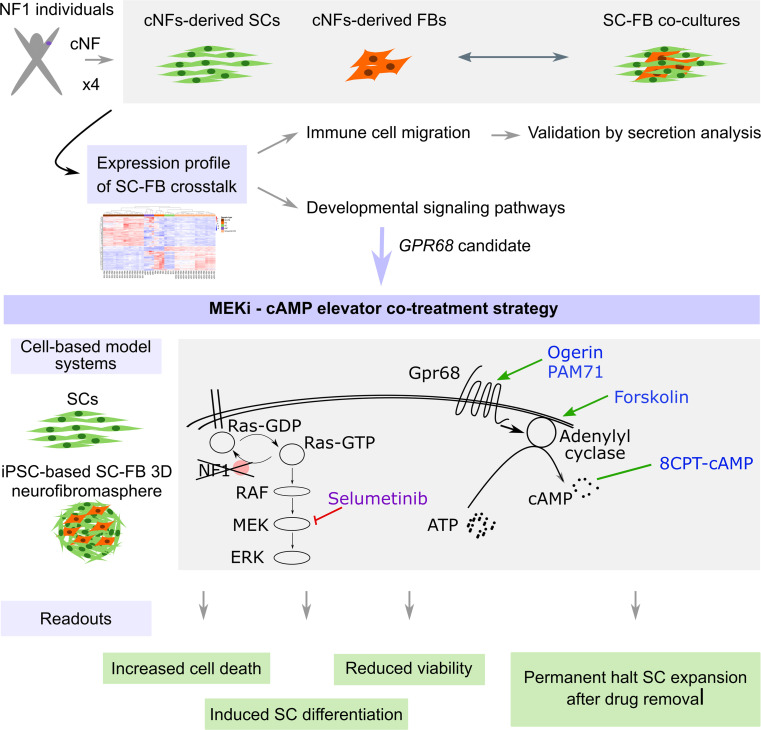

## Introduction

Cutaneous neurofibromas (cNFs) are benign Schwann cell (SC) tumors that most likely originate in the subepidermal glia (1) and form discrete nodules that bump out of the skin. cNFs normally appear during puberty and are present in more than 95% of patients with neurofibromatosis type 1 (NF1) (2). The number of cNFs present in each patient is greatly variable, ranging from tens to thousands, but their number always increases throughout life (3–5). cNFs might be itchy or painful but most are asymptomatic. Although cNFs do not progress to malignancy and rarely are associated with clinical complications, they can greatly impact the quality of life of individuals with NF1 due to disfigurement, dysesthesia, and the psychological effect of the perceived disease visibility (6, 7). Current clinical management of cNFs involves the surveillance of lesions that may require surgical resection (8, 9). The development of effective drugs for treating cNFs is currently an active research area in the field. Different MEK inhibitors (MEKis) are being assayed systemically and topically (10, 11). However, further development of new effective drugs and delivery methods is highly needed.

cNFs are composed of different cell types (12), including mostly SCs, which constitute between 40% and 80% of all cellular content (13, 14), and fibroblasts (FBs). cNFs also contain a rich immune infiltrate, composed of mast cells (15), lymphocytes, macrophages, and others. Endothelial cells, perineural cells, and axons are also present, all embedded in a collagen-rich extracellular matrix (16, 17). Only SCs bear a double inactivation of the *NF1* gene (18, 19), making them susceptible to overproliferation. However, evidence obtained in vitro and using genetically modified animal models illustrates an essential role of the rich *NF1*^+/–^ microenvironment in cNF (revised in ref. 20). Whether the *NF1*^+/–^ status of the microenvironment is essential or just a facilitator of cNF development is still under debate. In humans, sporadic plexiform neurofibromas carrying a biallelic inactivation of *NF1* have been reported in individuals with the rest of the cells being *NF1* WT cells (21). The most studied component of the microenvironment has been the immune infiltrate and its role in promoting neurofibroma growth (revised in ref. 22). The complex interactions that might exist between SCs and the immune system, and also the interactions between SCs and the remaining cell components (FBs, axons, etc.), need to be further clarified. cNF cell components can be dissociated and either sorted by flow cytometry or panning strategies (23) or by differential growing conditions in culture. In this regard, conditions for selectively expanding *NF1*^–/–^ SCs and *NF1*^+/–^ FBs from cNFs are well established (18).

Neurofibromin’s role in Ras/MAPK regulation is well characterized (24), as in its role in the context of SCs(25, 26). The role of neurofibromin in the cAMP pathway has also been determined (27, 28), although it is much less understood at the molecular level. Recently, cAMP intracellular elevation has been involved in the reduction of SC precursor self-renewal and cNF SC proliferation (29). In agreement, *NF1*^+/–^ and *NF1*^–/–^ cNF-derived SC cultures can be obtained basically by modifying the exposure to cAMP-elevating agents, because *NF1*^–/–^ SCs decrease proliferation after long exposures to elevated cAMP, compared with *NF1*^+/–^ SCs (18). This could be somehow related to the increased basal cAMP levels in *Nf1*-deficient mouse SCs compared with *Nf1*^+/–^ SCs (30). In addition, the level of cAMP signaling is crucial for switching from a proliferative state to a myelin-forming differentiation state (31). Both the Ras/MAPK and the cAMP/PKA pathways need to be active for prolific SC proliferation (32).

In the present work, we have investigated the crosstalk between cNF-derived *NF1*^–/–^ SCs and *NF1*^+/–^ FBs. We identified a transcriptional signature due to the SC-FB interaction and enriched in genes involved in immune cell migration and developmental signaling pathways. The identification of the cAMP elevator G protein–coupled receptor 68 (*GPR68*) prompted us to test the effect of elevating cAMP in *NF1*^–/–^ SCs, alone or in combination with the MEKi selumetinib. Results using primary cNF-derived SCs and validated in 3D neurofibromaspheres point to the concomitant inhibition of the Ras pathway and the activation of the cAMP pathway as a potential therapeutic strategy for cNFs.

## Results

**Figure 1:**
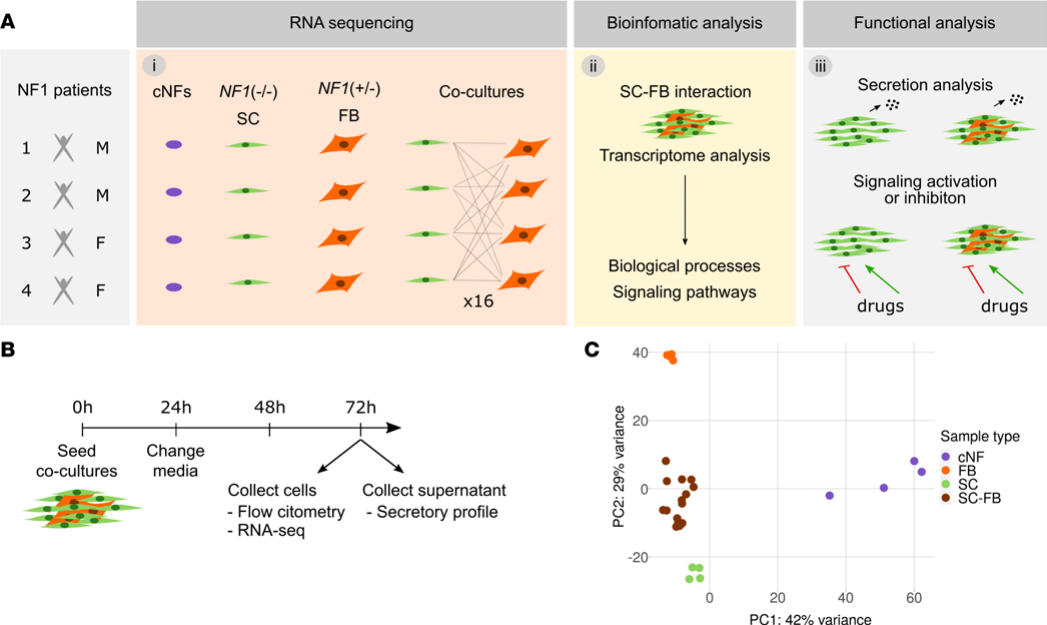
Experimental set up workflow. (**A**) Schematic representation showing the 3 different phases of the project: (i) establishment of SC and FB cultures from cNFs and establishment of SC-FB cocultures and RNA-Seq, (ii) bioinformatic analysis, and (iii) functional analysis. (**B**) Timeline of the coculture experiment. (**C**) Principal component analysis of cNF, SC, and FB single cultures and SC-FB cocultures from RNA-Seq data showing a clear separation of cell types. SC, Schwann cells; FB, fibroblasts; SC-FB, SC-FB cocultures; cNF, cutaneous neurofibroma.

We developed an experimental framework to investigate cNF-derived SC-FB crosstalk, based on the identification of transcriptional changes in both cell types specifically produced by their interaction (Figure 1A). We first identified both constitutional and somatic *NF1* mutations present in cNFs (Supplemental Table 1; supplemental material available online with this article; https://doi.org/10.1172/jci.insight.168826DS1) and set up experimental conditions to analyze the transcriptome of the SC-FB interaction.

### Transcriptome analysis of cNFs and cNF-derived cell cultures and cocultures.

We obtained cNFs from 4 individuals with NF1 (2 female and 2 male participants; Supplemental Table 1 and Methods) and derived single cultures of *NF1*^–/–^ SCs and *NF1*^+/–^ FBs from each tumor (18). We sequenced the entire *NF1* coding region (33) from these cultures and demonstrated their constitutional or somatic nature by comparing *NF1*-specific mutations in *NF1*^+/–^ FB cultures. We also applied microsatellite analysis for loss-of-heterozygosity identification in the *NF1* region (34). Among the distinct cNFs analyzed, we selected 4 cNFs from 4 different patients with NF1 (Supplemental Table 1), performed whole-exome sequencing of both cNF-derived SC and FB cultures, and analyzed all constitutional genomic variance present in coding genes as well as all somatic mutations present in *NF1*^–/–^ SCs. As previously reported (35), we did not identify any significant recurrently mutated gene present in NF1-associated cNF-derived SCs, besides the *NF1* gene.

Using single cultures of SCs and FBs derived from cNFs, we set up conditions for generating SC-FB cocultures, with a final defined proportion of cells resembling that present on average in neurofibromas, approximately 60%–70% SCs and 30%–40% FBs (36) (Supplemental Figure 1). We controlled cell density over time and assessed the presence of both cell types using different markers. To capture biological differences coming from the distinct cNFs, we generated 16 SC-FB cocultures by mixing each SC culture with FBs derived from each of the 4 cNFs (Figure 1A). Both single cultures and cocultures were submitted to experimental conditions (Figure 1B), and, after 72 hours, cells and supernatants were collected. We used some of the harvested cells to quantify the percentage of both cell types through flow cytometry analysis using the SC maker p75 (*NGFR*), thus keeping track of the exact proportions in single cultures and cocultures (Supplemental Figure 2). We extracted RNA and performed RNA-Seq for all 16 SC-FB cocultures, the 4 single SC cultures, the 4 single FB cultures, and the 4 original cNFs. Principal component analysis (Figure 1C) nicely grouped samples of each type and separated the different groups of samples. The two main components capturing most parts of the variability among samples found similarities and differences between SC-FB cocultures and single cultures as well as between SC-FB cocultures and cNFs.

### An expression profile of SC-FB crosstalk.

**Figure 2:**
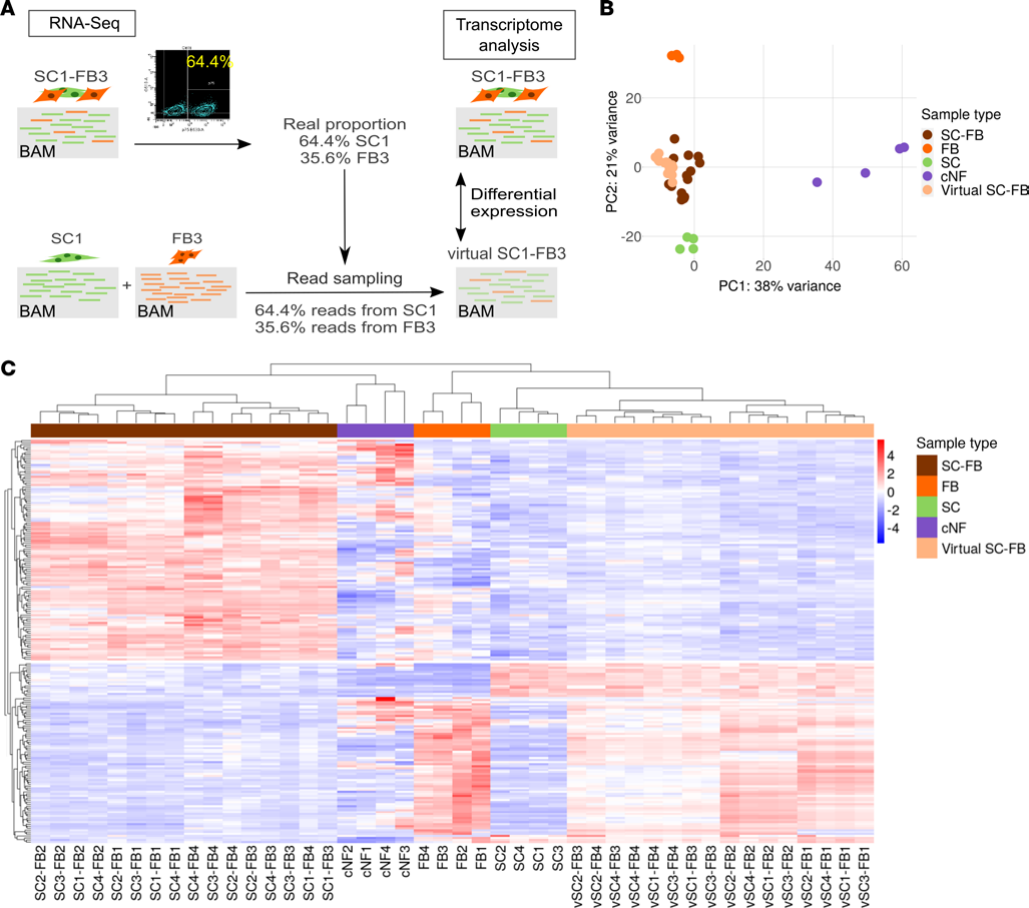
Transcriptome analysis of cNFs, cNF-derived single cultures, and SC-FB cocultures identifies expression profiles due to SC-FB interactions. (**A**) Schematic representation of in silico SC-FB coculture approach for generation of virtual cocultures. Virtual cocultures were generated by random sampling of a number of reads from the BAM files of the single cultures and mixing samples from each single culture with an exact proportion of cocultured samples. (**B**) Principal component analysis of cNF, SC, and FB single cultures; SC-FB cocultures; and virtual SC-FB cocultures for whole-genome RNA-Seq, showing the proximity between real and virtual cocultures. (**C**) Heatmap plot representing an unsupervised cluster analysis of differentially expressed genes (adjusted *P* < 0.05) between real and virtual SC-FB cocultures. SC, Schwann cells; FB, fibroblasts; SC-FB, SC-FB cocultures; cNF, cutaneous neurofibroma.

**Figure 3:**
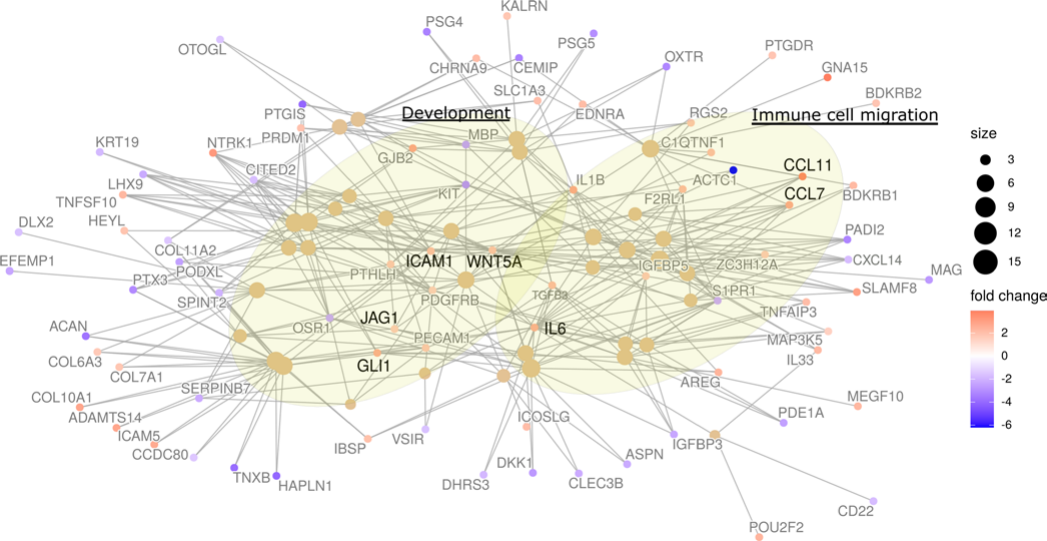
Network analysis of biological processes captured by the differential upregulated genes in real versus virtual SC-FB cocultures. Network plot showing the most differentially upregulated genes and the biological processes (BP) captured by them. Lines define shared genes among the distinct BPs. The size of the sphere that represents the BP is proportional to the number of genes contained.

From all genes expressed in SC-FB cocultures, we aimed to identify a transcriptional signature specifically representing genes whose expression was dominated by the interaction between SCs and FBs. For that, we set up a specific bioinformatic analysis (Figure 2A). For each of the 16 SC-FB cocultures, a virtual companion coculture was generated by the random sampling of reads from the 2 SC and FB single cultures used for each coculture generation. For instance, for the coculture SC1-FB3, the random sampling of reads was performed using RNA-Seq data from single SC1 and FB3 cultures. The proportion of reads sampled from every single culture was equivalent to the exact proportion of SCs and FBs present in each coculture, determined by flow cytometry analysis (Supplemental Figure 2). In this regard, companion virtual cocultures could be seen as cocultures with no expression changes due to SC-FB interactions, containing the same expression in SCs and FBs as in their respective single cultures, although with a mixed composition. Principal component analysis revealed a high degree of similarity in expression between real and virtual cocultures (Figure 2B), although it also showed a consistent expression differences between cocultures. To identify an expression profile specific to SC-FB crosstalk, a differential expression analysis between real and virtual cocultures was performed, and results were plotted in a heatmap representing an unsupervised cluster analysis of differentially expressed genes (Figure 2C). This analysis identified a transcriptional signature containing upregulated and downregulated genes in real versus virtual cocultures (Supplemental Data File 1). To validate this expression profile in a different neurofibroma model system, we selected all upregulated genes and analyzed their expression in an induced pluripotent stem cell–derived (iPSC-derived) 3D neurofibroma model, consisting of spheroids containing only SCs or containing an admixture of SCs and FBs (37). An unsupervised cluster analysis of this upregulated gene signature in real and virtual cocultures, SCs and SC-FB spheroids, and cNFs indicated that most genes of the signature were also specifically expressed in SC-FB spheroids, clustering together with real SC-FB cocultures (Supplemental Figure 3). In comparison, only a small part of the genes present in the identified expression profile were expressed in spheroids made only of SCs, which clustered together with virtual cocultures (Supplemental Figure 3). Thus, we identified a robust SC-FB crosstalk signature that was preserved in different neurofibroma coculture systems. We next aimed to translate the identified SC-FB crosstalk signature into biological processes and signaling pathways. For that, we selected genes constituting the signature and performed an enrichment analysis (Figure 3 and Supplemental Data File 2). We selected two groups of biological processes: (a) those related to immune response, immune cell migration, and chemotaxis and (b) those broadly grouped as commonly involved in developmental processes and developmental signaling pathways (Figure 3).

### SC-FB interaction elicits the secretion of multiple cytokines and chemokines involved in immune cell migration.

**Figure 4:**
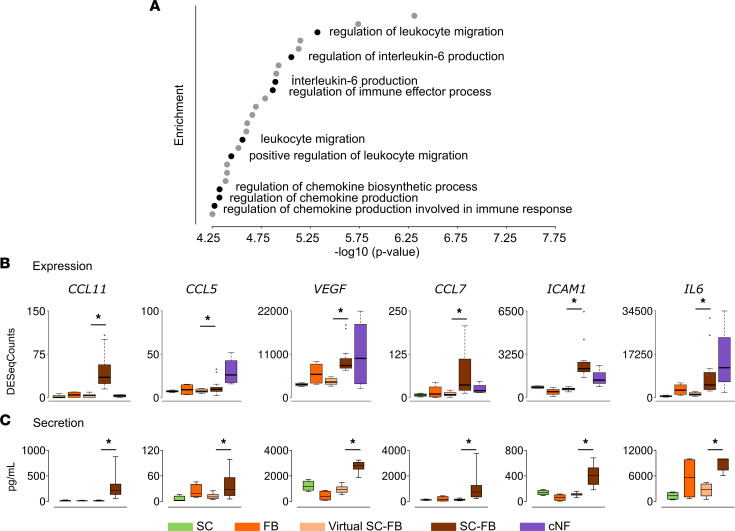
The SC-FB interaction elicits the secretion of multiple cytokines and chemokines involved in immune cell migration. (**A**) Enrichment analysis of upregulated genes in real cocultures, showing the 25 most significant biological processes (BPs). BPs related to cytokine and chemokine production and immune cell migration are shown in black. (**B** and **C**) Correlation between expression data from RNA-Seq (**B**) and secretion data from Luminex (**C**) of several upregulated genes in SC-FB coculture experiments compared with single cultures and primary tumors. Data represent mean ± SEM from 4 independent SC and FB cultures, 16 independent SC-FB virtual and real cocultures, and 4 independent cNFs. One-tailed paired *t* test (*P* ≤ 0.05) between virtual and real cocultures. SC, Schwann cells; FB, fibroblasts; virtual SC-FB, virtual SC-FB cocultures; SC-FB, SC-FB cocultures; cNF, cutaneous neurofibroma.

Among the twenty-fifth most significantly enriched biological processes, 9 were related to cytokines and chemokines influencing immune cell regulation and migration (Figure 4A). We selected some of the genes representing these biological processes, for example, CCL5, CCL7, CCL11, ICAM1, NCAM, VEGF, and IL-6, and performed an expression analysis using RNA-Seq data, considering SC and FB single cultures, real and virtual cocultures, and cNFs (Figure 4B). All genes showed statistically significant expression differences between real and virtual cocultures, which were mainly upregulated in real cocultures. To investigate whether transcription differences were consistent at the protein level and to investigate their potential paracrine action, we measured the secretion of their protein products in the supernatants of all single SC and FB cultures and all cocultures using Luminex (see Methods and Supplemental Data File 3). Again, we generated a companion virtual coculture for each of the 16 cocultures, similarly to the way described in Figure 2A, but this time we considered the selected protein products being measured in single SC and FB culture supernatants and mixed them according to the real SC-FB proportions in each real coculture. All secreted products of selected genes were also significantly overexpressed in real versus virtual cocultures (Figure 4C), indicating a correlation between gene transcription (RNA-seq data) and protein secretion (secretion analysis by Luminex). Thus, these results indicated that signaling for immune cell recruitment in cNFs is at least partially produced by the SC-FB crosstalk.

### Gpr68 activation by ogerin reduces viability and proliferation of cNF-derived primary SCs in both single cultures and SC-FB cocultures.

**Figure 5:**
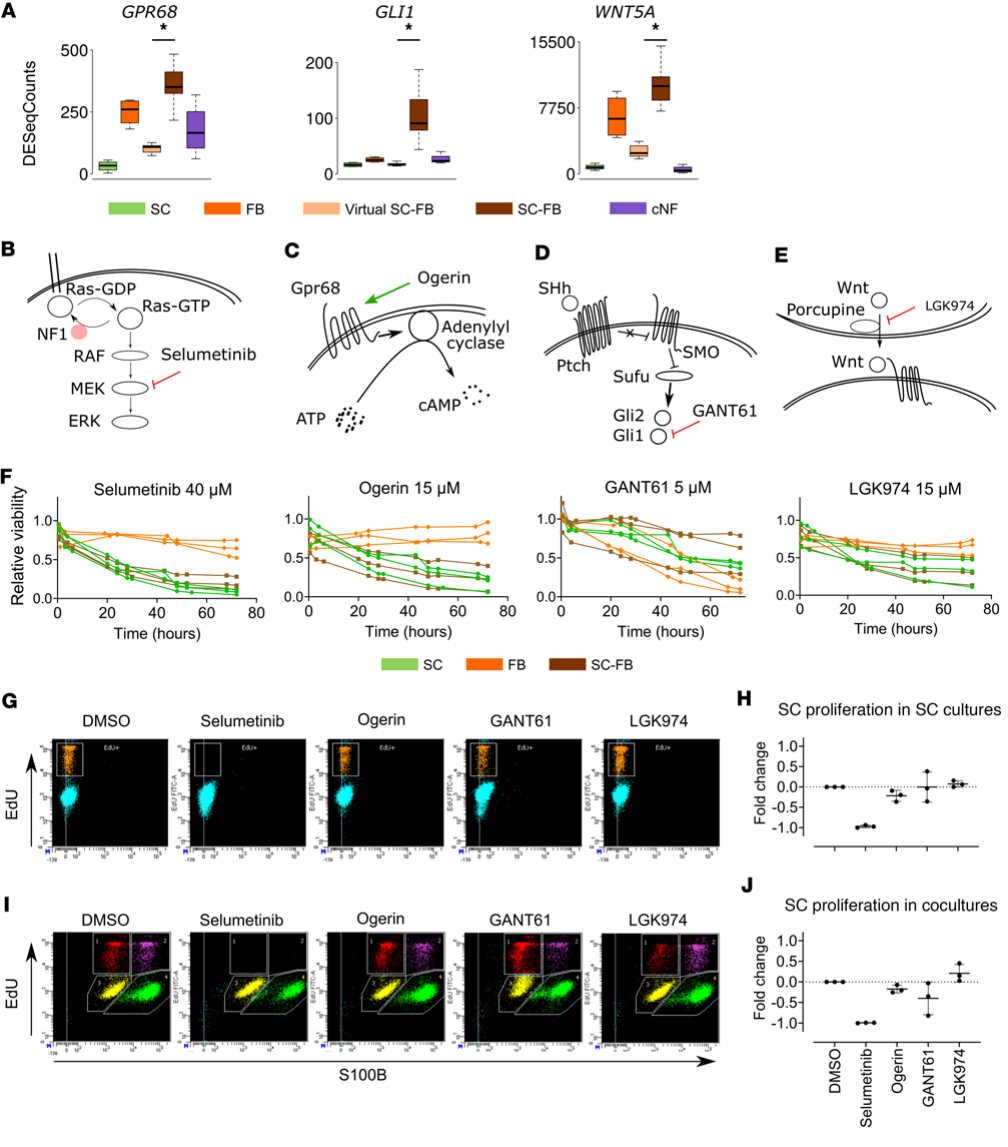
Ogerin decreases SC proliferation and viability in cNF-derived SCs and cocultures. (**A**) Expression data from RNA-Seq of *GPR68*, *GLI1*, and *WNT5A*. Data represent mean ± SEM from 4 independent SC and FB cultures, 16 independent SC-FB virtual and real cocultures, and 4 independent cNFs. One-tailed paired *t* test (*P ≤ 0.05) between virtual and real cocultures. (**B**–**E**) Mechanism of action of selected drugs, (**B**) selumetinib, (**C**) ogerin, (**D**) GANT61, and (**E**) LGK974, in the context of their signaling pathways. (**F**) Effect of selected drug treatments on cell viability in primary SC (green), FB (orange), and SC-FB cocultures (brown). Cell viability was monitored over 72 hours using the RealTime-Glo MT Cell Viability Assay. Three different primary samples were used per cell type. Mean is represented. Data are expressed as relative viability to DMSO-treated control cells. (**G** and **H**) Effect of drug treatments on cell proliferation in primary SC cultures. After 48 hours of treatment, cell proliferation was assessed by the Click-iT EdU Flow Cytometry Assay. (**G**) Representative flow cytometry plots of the different treatments of 1 primary SC culture. (**H**) Data are expressed as mean fold change normalized to DMSO-treated control cells ± SEM from 3 different primary SC cultures. (**I** and **J**) Effect of drug treatments on cell proliferation in SC-FB cocultures. After 48 hours of treatment, cell proliferation was assessed by the Click-iT EdU Flow Cytometry Assay in combination with S100B staining to distinguish the SC population. (**I**) Representative flow cytometry plot of the different treatments of 1 SC-FB coculture. (**J**) Data are expressed as mean fold change normalized to DMSO-treated control cells ± SEM from 3 different primary SC cultures. SC, Schwann cells; FB, fibroblasts; virtual SC-FB, virtual SC-FB cocultures; SC-FB, SC-FB cocultures; cNF, cutaneous neurofibroma.

We selected a second group of biological processes broadly grouped as involved in developmental processes and developmental signaling pathways. We chose different genes central to these biological processes (Figure 5 and Supplemental Table 2), including JAG1 (Notch signaling), GLI1 (Sonic Hedgehog signaling); WNT5A (Wnt signaling); TGFb3 (TGF-β signaling); AREG (EGF/TGF-α signaling); TGFA (TGF-α signaling); and GPR68 (cAMP signaling). To identify whether any of these pathways had an effect on cNF growth, we selected compounds that increase or decrease the function of their protein products and performed a preliminary cell viability screening assay using human cNF-derived SC cultures (data not shown). From the results obtained, we selected *GPR68*, *GLI1*, and *WNT5A* as potential targets to further characterize their effect in SC cultures and SC-FB cocultures. Expression analysis showed that these 3 genes were significantly overexpressed in real versus virtual cocultures (Figure 5A). Three different compounds were selected to modulate their gene products: ogerin, an allosteric activator of the Gpr68, which, in turn, activates adenylyl cyclase and elicits the production of cAMP (Figure 5C); GANT61, an inhibitor of the transcription factor 1 (GLI1, Figure 5D); and LGK974, an inhibitor of the secretion of Wnt via Porcupine (Figure 5E). We used selumetinib, a MEKi authorized by the FDA and EMA to treat inoperable plexiform neurofibromas (pNFs) in children (38), as a positive control (Figure 5B). Functional analyses performed and human cNF-derived SCs used for each analysis are summarized in Supplemental Table 3. We tested the toxicity of different concentrations of selected drugs using skin FBs derived from patients with NF1 (Supplemental Figure 4) and selected 1 dose for further experiments (15 μM ogerin, 5 μM GANT61, and 15 μM LGK974). Next, we characterized the physiological role of these signaling pathways in SC and FB single cultures and SC-FB cocultures. Cells were seeded, and, after 24 hours, media with the different drugs or vehicle (DMSO) was replaced. We evaluated cell viability through the following 72 hours using a luminometric assay (RealTime-Glo; Figure 5F) and cell proliferation at 48 hours using flow cytometry (Click-iT EdU; Figure 5, G–J) in cultures from 3 independent cNFs. Selumetinib treatment strongly reduced cell viability in SC cultures and SC-FB cocultures with a slight effect on FB cultures (Figure 5F). A similar viability pattern was observed in ogerin-treated SC cultures, alone or in cocultures, with no effect on FBs viability. For GANT61 and LGK974, although they exhibited a certain effect on SC viability, the response was either not as clear as that from ogerin (GANT61) or exhibited inconsistencies among different cNF-derived cells (LGK974). Furthermore, selumetinib elicited a potent arrest of SCs in single cultures after 48 hours (Figure 5, G and H). Using S100B staining in combination with Click-iT EdU we measured the proliferation status of SC and FB populations in cocultures (Figure 5, I and J). Again, selumetinib elicited a strong arrest of both SCs and FBs in cocultures. Ogerin produced a slight arrest on SCs, in both single and cocultures, that was consistent among cultures derived from the 3 independent cNFs. For GANT61 and LGK974, responses were either with no effect (LGK974) or inconsistent among biological replicates (GANT61).

### Selumetinib-ogerin cotreatment induces loss of viability, differentiation, and death of cNF-derived primary SCs.

**Figure 6:**
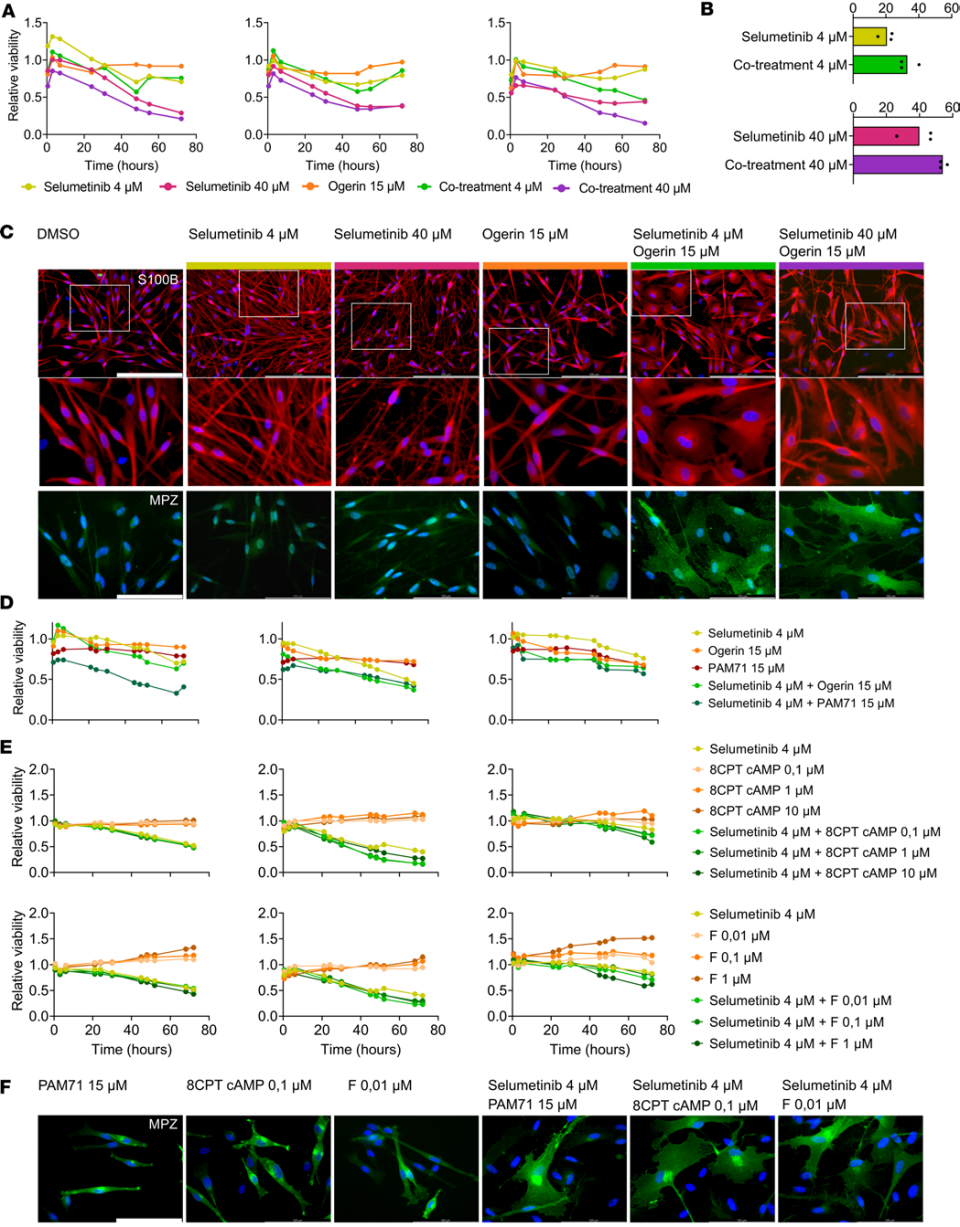
Selumetinib-ogerin cotreatment leads to differentiation and increased cell death in cNF-derived primary SCs. (**A**) Effect of selumetinib and ogerin treatments and cotreatments on cell viability in cNF-derived primary SC cultures. Cell viability was monitored over 72 hours using the RealTime-Glo MT Cell Viability Assay. Three different SC cultures were used. Mean is represented, and data are expressed as relative viability to DMSO-treated control cells. (**B**) Effect of selumetinib treatments and selumetinib-ogerin cotreatments on cell death in cNF-derived primary SC cultures. Cell death was monitored using the flow cytometry Annexin V Apoptosis Detection Kit. Data are expressed as the percentage of cells in early and late apoptosis per condition in 3 different SC cultures. Median is represented. One-tailed unpaired *t* test (*P* ≤ 0.05). (**C**) Immunocytochemical analysis for S100B and MPZ in SC-treated cultures. DAPI was used to stain cell nuclei. Scale bars: 200 μm (S100B), except in higher-magnification views; 100 μm (MPZ). (**D**) Effect of selumetinib and ogerin or the ogerin-analog PAM71 treatment and cotreatment on cell viability in cNF-derived primary SC cultures. Three SC cultures were used. Mean is represented, and data are expressed as relative viability to DMSO-treated control cells. (**E**) Effect of selumetinib and the cAMP analog 8CPT (top) or forskolin (F; bottom) on cell viability in cNF-derived primary SC cultures. Three different SC cultures were used. Mean is represented, and data are expressed as relative viability to DMSO-treated control cells. (**F**) Immunocytochemical analysis for MPZ in treated SC cultures. DAPI was used to stain cell nuclei. Scale bars: 100 μm.

Results regarding plexiform neurofibroma treatment with MEKis (38–40) opened the possibility of using these drugs for cNFs, either alone or potentially combined with other agents. Given the role of Ras and cAMP pathways in the balance of SC proliferation/differentiation (31, 32), we hypothesized that unbalancing both pathways with the selumetinib-ogerin cotreatment could serve to prevent *NF1*^–/–^ SC proliferation in cNFs. To test this possibility, we confirmed that ogerin treatment was elevating cAMP levels in cNF SCs (Supplemental Figure 5). Then, we examined the toxicity effect of selumetinib-ogerin cotreatment on NF1 skin-derived FBs and monitored viability using RealTime-Glo for 72 hours (Supplemental Figure 6). This cotreatment elicited no toxicity on skin FBs using either low or high selumetinib concentrations. Next, we tested the cotreatment effect on *NF1*^–/–^ SC viability in 3 independent human cNF SC cultures, employing the same assay (Figure 6A). Selumetinib-ogerin cotreatment, using either low or high concentrations of selumetinib, enhanced the loss of viability of SCs compared with single agents alone. We observed that in cotreatment conditions SC cultures showed a high amount of cell debris, and the morphology of many SCs changed, so we broadened the analysis of SC physiological state. We measured apoptosis using flow cytometry analysis (Figure 6B and Supplemental Figure 7). Ogerin alone almost had no effect on SC apoptosis, but ogerin-selumetinib cotreatment enhanced the degree of apoptosis compared with selumetinib as a single agent, both at low and high concentrations of the MEKi. Furthermore, we carefully characterized the effect of treatments on SC morphology. S100B staining showed that, while ogerin alone elicited no apparent morphological change in SCs, selumetinib, especially at high concentrations, enhanced the rectilinear and spindle shape morphology of SCs, decreasing the cytoplasmic volume (Figure 6C). In contrast, selumetinib-ogerin cotreatment made SCs spread and flatten and expanded the cell membrane (Figure 6C). To rule out whether the morphological change of cotreated SCs reflected SC differentiation and myelinization, we immunostained these cells using a myelin protein zero (MPZ) antibody (Figure 6C). While cells treated with single agents alone did not stain for MPZ protein, cotreated cells were highly MPZ positive, indicating a promyelinization-differentiating SC phenotype. To explore whether the observed physiological changes were specific to the GPR68 activation and were produced by the activation of the cAMP pathway, we substituted ogerin either by another GPR68 activator (positive analog modulator 71 [PAM71], ref. 41, Figure 6D) or directly by cAMP analogs/adenylyl cyclase activators (8CPT cAMP and forskolin, Figure 6E and Supplemental Figure 8). Selumetinib-PAM71 cotreatment triggered the same loss of SC viability as selumetinib-ogerin (Figure 6D). The combinations composed of 8CPT cAMP–selumetinib and forskolin-selumetinib exhibited a similar decrease in SC viability (Figure 6E), mainly driven by selumetinib. However, as well as for ogerin-selumetinib combination, in all cotreatments we observed a change in SC morphology. MPZ staining of all the different cotreatments showed that, while PAM71 or low concentrations of 8CPT cAMP and forskolin alone elicited no change in MPZ expression or morphological change, all combinations with selumetinib induced a clear promyelinization state in SCs (Figure 6F). This result indicated that the SC differentiation phenotype was mainly driven by cAMP elevation, but being dependent on the degree of Ras/MAPK pathway activation. Altogether, ogerin-selumetinib cotreatment induced loss of viability, SC myelinization, and death of human cNF-derived primary SCs.

### Selumetinib-ogerin cotreatment induces sphere disaggregation, cell death, and loss of viability in an iPSC-derived 3D neurofibromasphere model.

**Figure 7:**
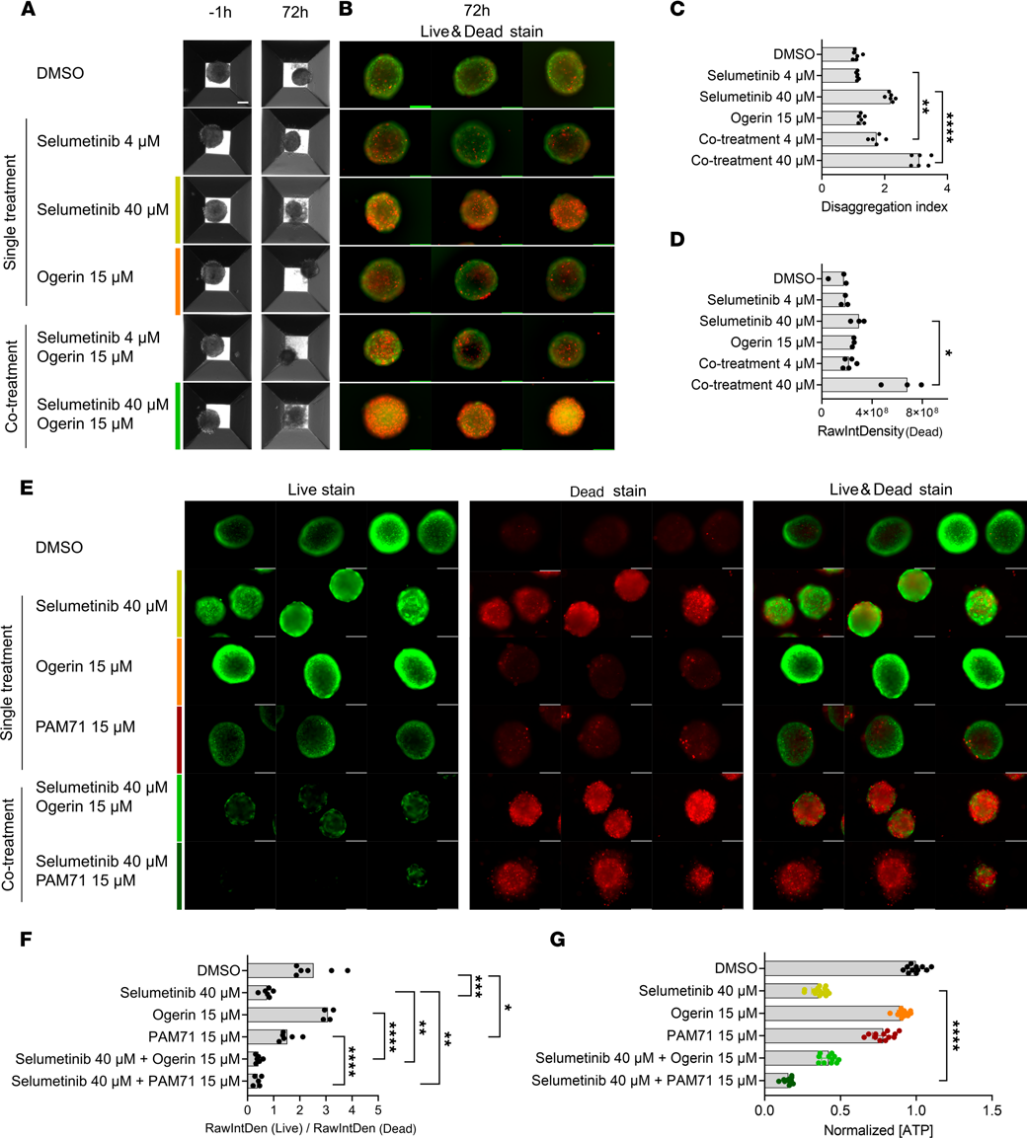
Selumetinib-ogerin and selumetinib-PAM71 cotreatments increase cell death in neurofibromaspheres. (**A**) Representative phase-contrast images showing neurofibromasphere morphology differences at –1 hour (nontreated) and at 72 hours after treatment with DMSO-treated (vehicle) spheroids, single treatment, and cotreatments. Neurofibromapheres were generated from *NF1*^–/–^ iPSC-derived differentiating SCs mixed with human cNF-derived FBs. Scale bar: 100μm. (**B**) Representative images of Acridine Orange– (ThermoFisher) (live, green) and propidium iodide–stained (dead, red) neurofibromaspheres after 72 hours of drug treatment. Scale bars: 100 μm. (**C**) Disaggregation index (DI) of neurofibromaspheres in **A**, showing a higher effect in cotreatments than in single treatments. DI is calculated by measuring disaggregated spheroid area normalized by spheroid area at 72 hours of drug treatment. Data are shown as the median of *n* ≥ 5 spheroids. One-tailed unpaired *t* test (**P ≤0.01 and ****P ≤ 0.0001, comparing 4 μM selumetinib with 4 μM cotreatment or 40 μM selumetinib with 40 μM cotreatment). (**D**) Raw integrity density of death channel of neurofibromaspheres in **B**, showing a higher effect in cotreatments than in single treatments. Data are shown as the median of *n* ≥ 3 spheroids. One-tailed unpaired *t* test (*P ≤ 0.05, comparing 4 μM selumetinib with 4 μM cotreatment or 40 μM selumetinib with 40 μM cotreatment. (**E**) Representative images of Acridine Orange (live, green), propidium iodide (dead, red) and merge (live and death) at 72 hours after selumetinib and ogerin or PAM71 treatments and cotreatments. (**F**) Ratio of raw integrity density (RawIntDen) in live and death channels of neurofibromaspheres in **E**. Data are shown as the median of *n* ≥ 5 spheroids. One-tailed unpaired *t* test performing a Holm-Bonferroni post hoc adjustment (*P ≤ 0.05, **P ≤0.01, ***P ≤ 0.001, and ****P ≤ 0.0001). (**G**) Effect of selumetinib, ogerin, and PAM71 treatment and cotreatment on cell viability in neurofibromaspheres. Cell viability was measured at 72 hours after treatment using Cell Titer Glo 3D viability assay. Two independent experiments were performed and at least 5 spheroids per condition/experiment were used. One-tailed unpaired *t* test (****P ≤ 0.0001) comparing 40 μM selumetinib with 40 μM selumetinib-ogerin cotreatment or 40 μM selumetinib with 40 μM selumetinib-PAM71 cotreatment. FB, fibroblast; iPSC, induced pluripotent stem cell; NC, neural crest; SC, Schwann cell.

We next validated selumetinib-ogerin cotreatment results in an independent neurofibroma model that we recently developed (37). This model consists of the generation of neurofibromaspheres, composed of human iPSC-derived *NF1*^–/–^ differentiating SCs (~70%) mixed with human neurofibroma-derived primary FBs (~30%). Upon engraftment in the sciatic nerve of nude mice, these neurofibromaspheres generate genuine human neurofibroma-like tumors, with histology recapitulating most features of human neurofibromas (37). For this experiment, we used neurofibromaspheres containing cNF-derived FBs together with iPSC-derived *NF1*^–/–^ differentiating SCs (Supplemental Figure 8). Single treatment and cotreatments were evaluated after 72 hours. At this point, spheres under selumetinib-ogerin cotreatment conditions lost compactness and exhibited an enhanced disaggregated appearance compared with spheres under single treatment and controls conditions (Figure 7A). We quantified disaggregation and generated a disaggregation index (see Methods; Figure 7C). Selumetinib-ogerin cotreatments exhibited a significantly enhanced disaggregation index compared with single agents and controls. Furthermore, by using a life and death staining assay, we observed a significant increase in cell death in selumetinib-ogerin cotreatments, compared with single treatments alone, especially at high concentrations of selumetinib (Figure 7, B and D). Thus, selumetinib-ogerin cotreatment response on neurofibromaspheres was consistent with results obtained using human cNF-derived primary SC cultures. As we did for primary SC cultures, we substituted ogerin with PAM71. We observed a very similar effect on neurofibromasphere cell death using both selumetinib-ogerin and selumetinib-PAM71 cotreatments (Figure 7E), as assessed by live and death assay. We quantified this assay (Figure 7F) that showed a significant decrease of living cells in both cotreatments compared with selumetinib alone, supporting the GPR68 specificity in this physiological change. In addition, we analyzed sphere viability using the Cell Titer Glo 3D viability assay and observed that selumetinib-PAM71 cotreatment also elicited a significant decrease in cell viability when compared with selumetinib treatment alone (Figure 7G). Altogether, these results support the possibility of using different agents to activate GPR68 in combination with selumetinib to exert a detrimental effect on neurofibromas.

### Ogerin-selumetinib cotreatment elicits a permanent halt on cNF-derived SC culture expansion.

**Figure 8:**
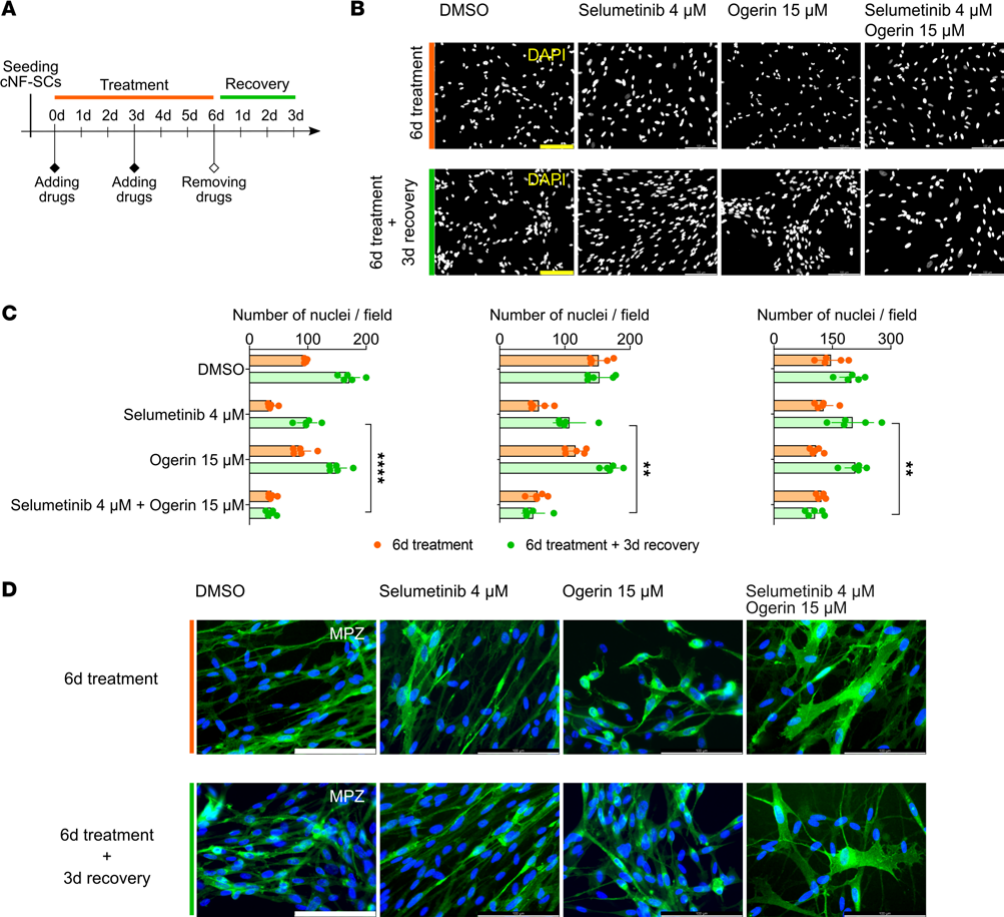
Selumetinib-ogerin cotreatment elicits a permanent halt on cNF-derived primary SC cultures. (**A**) Schematic of the experimental design. (**B**) Representative images of DAPI-stained nuclei of treated SC cultures at 6 days of treatment (top) and 6 days of treatment plus 3 days of recovery (bottom). Scale bar: 100 μm. (**C**) Quantification of DAPI-stained nuclei per field of 3 independent treated SC cultures at 6 days of treatment (orange) and 6 days of treatment plus 3 days of recovery (green). One-tailed unpaired *t* test (**P ≤0.01 and ****P ≤ 0.0001) comparing 4 μM selumetinib with 4 μM cotreatment. (**D**) Representative images of immunocytochemical analysis for MPZ in treated SC cultures at 6 days of treatment (top) and 6 days of treatment plus 3 days of recovery (bottom). DAPI was used to stain cell nuclei. Scale bars: 100 μm.

Inoperable pNFs in NF1 children might progress if treatment concentrations of MEKi are reduced or discontinued (40, 42). Our results indicated that selumetinib-ogerin cotreatment in cNF-derived primary SC cultures reduced viability, increased cell death, and induced SC differentiation compared with selumetinib treatment alone. Thus, we reasoned that cotreatment could be imposing a permanent physiological change on SCs and designed an experiment to monitor cell recovery after treatment (Figure 8A). We submitted primary SCs derived from 3 cNFs from independent patients 6 days after treatment with selumetinib or ogerin alone or a combination of both. After treatment, cells were cultured for 3 more days with media without drugs. Cell recovery was analyzed by counting the cells at 6 days of treatment and at the end of the experiment (Figure 8B). MPZ staining was performed to asses myelination/differentiation. We observed differences in the number of cells remaining in cultures after selumetinib and ogerin treatment alone or in combination compared with those after DMSO control treatment (Figure 8C), an effect mainly driven by selumetinib. However, after 3 days of recovery with no drugs, SCs treated with selumetinib or ogerin alone resumed cell proliferation and culture expansion, similar to control conditions. In clear contrast, the selumetinib-ogerin combination elicited a permanent halt of SC expansion and even decreased cell numbers in the 3 independent cNF SC cultures (Figure 8C). The number of cells was significantly reduced after the recovery period, and a differentiation phenotype was adopted by many SCs (Figure 8D), a physiological change not observed in any other condition tested. These results clarified the cotreatment effect on human cNF-derived SC cultures, which was not seen in SCs treated with selumetinib alone, and are supportive of a combination of MEKi and cAMP elevators as a potential treatment for cNFs.

## Discussion

We set up a robust experimental framework that was carefully executed to capture the crosstalk between SCs and FBs at a transcriptome level. The transcriptome of 16 SC-FB cocultures was analyzed and compared with that of their corresponding single cultures and original cNFs to minimize the potential biological biases of particular cNFs or cell types. Indeed, unsupervised cluster analysis of the transcriptome of SC-FB cocultures evidenced the dominant influence of FBs over SCs in the whole expression signature (Figure 2C). The identified SC-FB crosstalk signature representing overexpressed genes was also mostly preserved in an iPSC-derived mixed SC-FB neurofibromasphere model (37) and not in SC-only spheres, strongly supporting the SC-FB interaction nature of the obtained expression signature. Enrichment analysis of this signature highlighted the presence of biological components related to immune response, immune cell migration, and chemotaxis and also signaling pathways important in developmental processes.

The most studied component of the cNF microenvironment has been the immune infiltrate (revised in ref. 22). Different molecules have been implicated in the recruitment of different cell types (mast cells, macrophages, T cells, dendritic cells) to neurofibromas: CCL2/CCL12/CCR2 (43); CSF1 (44); CXCL10/CXCR3 (45); IL-1b (23); IL-6 (46); and KITLG (SCF) (47, 48). In the present work, we identified some of these genes to be transcriptionally upregulated owing to the SC-FB interaction (CCL2, CXCL10, IL1B, IL-6), while others were not (CSF1) or were upregulated to a lesser extent (CCL5, KITLG). CSF1 and KITLG were highly expressed in FBs. At the protein level, secreted in supernatants of SC-FB cocultures, we confirmed the upregulated secretion of IL-6, CCL5, and other factors, such as CCL11, CCL7, and ICAM, and angiogenic factors, such as VEGF (Supplemental Data File 3). In this regard, our work shows that at least some of the secretion in proteins that promote immune cell recruitment into cNFs is specifically due to the interaction between SCs and FBs.

Components of signaling pathways involved in developmental processes were also enriched, such as JAG1 (Notch), GLI1 (Sonic Hedgehog); WNT5A (Wnt); TGFb3 (TGF-β); AREG (EGF/TGF-α); TGFA (TGF-α); and GPR68 (cAMP). After different methods of testing the physiological impact of these components, we selected the receptor GPR68 as an interesting target for cNF biology and/or treatment. We used ogerin, an allosteric modulator of GPR68 that potentiates proton activity and activates adenylyl cyclase, to activate GPR68. Upon ogerin treatment, cNF-derived SCs increased the levels of cAMP (Supplemental Figure 5), decreased SC viability, and slightly decreased SC proliferation in single cultures and SC-FB cocultures. In contrast, ogerin had no effect on cNF-derived FBs, or FBs derived from the skin of an individual with NF1.

The discovery of MEKis to treat plexiform neurofibromas has opened the possibility of using these drugs likewise for cNFs (38–40). However, despite effectiveness, results with pNFs and results using the *Nf1*-KO model (*Prss56*^Cre^, *Nf1*^fl/fl^) (11) for cNFs indicate that MEKi alone might not be a complete solution for mature cNFs, and the combination of MEKi with other agents emerged as a logical next step for cNF treatment. Neurofibromin plays a role in SCs and neurofibromas by regulating both the Ras/MAPK pathway (25, 26) and the cAMP pathway (18, 29, 30). Both pathways need to be active for SC proliferation (32). In fact, we collected evidence that the regulation of both pathways by neurofibromin could be coordinated (49). However, it is known that in SCs the level of cAMP signaling is crucial for switching from a proliferative state to a myelin-forming differentiating state (31). Thus, with all this information and the results obtained from exposing cNF-derived SCs to ogerin, we reasoned that unbalancing both pathways by the simultaneous combination of decreasing the RAS pathway and increasing the cAMP pathway could be a good strategy for preventing *NF1*^–/–^ SC proliferation and inducing differentiation and could potentially become a therapeutic strategy for cNFs. We tested this idea by performing selumetinib-ogerin cotreatments in cNF-derived primary SCs. This combination reduced cell viability, elicited a promyelinization differentiating SC phenotype, and enhanced apoptosis of SCs compared with single selumetinib treatment.

Ogerin has already been used in vitro and in vivo (50) although further information regarding off-target effects, in vivo toxicity, and kinetics, is required. Other GPR68 activators have been developed, such as the potent allosteric activator (~30 fold) termed MS48107 (or PAM71) (41) or 3 positive allosteric peptides [osteocrin_33–55_, CART(42–89)_9–28_, and corticotropin_17–40_] (51), providing alternatives to the use of ogerin for the activation of GPR68. To support the specificity of GPR68 in the physiological changes produced by ogerin, we also tested PAM71 alone or in combination with selumetinib. We observed that selumetinib-PAM71 cotreatment reduced viability and induced differentiation of cNF-derived primary SCs as well as the selumetinib-ogerin combination. In addition, to assess the degree of involvement of the cAMP pathway in the response elicited by GPR68 activation, we substituted ogerin with cAMP analogs and adenylyl cyclase activators (8CPT cAMP and forskolin, respectively). Again, we observed that low concentrations of these agents in combination with selumetinib produced similar physiological changes to cNF-derived primary SCs. These results represent the first in vitro data to our knowledge on the effect of selumetinib alone or in combination using human cNF-derived primary SCs.

Furthermore, we validated these results in the iPSC-derived 3D neurofibromasphere model (37). Selumetinib-ogerin cotreatment increased sphere disaggregation and cell death compared with each agent alone. We also substituted ogerin with PAM71. Selumetinib-PAM71 cotreatment induced cell death to the same extent as the selumetinib-ogerin combination, a result that was ratified by independently measuring sphere viability, because selumetinib-PAM71 significantly reduced neurofibromasphere viability compared with selumetinib alone.

Importantly, while selumetinib alone had a large effect on loss of viability in cNF-derived primary SC cultures and 3D neurofibromapheres, it did not induce a differentiation phenotype in cNF primary SCs or induce cell death to the same extent as selumetinib-ogerin cotreatment. These results suggested that this cotreatment could be imposing a permanent physiological change on SCs that selumetinib alone was not able to do. We answered this question by performing an experiment analyzing the recovery of cNF primary SCs after 6 days of treatment. SCs treated with selumetinib or ogerin alone resumed cell proliferation and culture expansion, while cultures treated with the selumetinib-ogerin combination showed a reduced the cell number, and many of the SCs adopted a promyelinating phenotype.

Overall, these results strongly support the combination of a MEKi and cAMP elevator as a potential therapy for cNFs. They open the possibility of using distinct cAMP elevators, even independently of GPR68 activation.

In summary, we identified GPR68 as one of the upregulated genes within a transcriptional signature specifically produced by the interaction of cNF-derived SCs and FBs in cocultures. The GPR68 allosteric activators ogerin and PAM71 in combination with the MEKi selumetinib reduced viability and induced the differentiation and death of cNF-derived primary SCs. These results were corroborated in an iPSC-derived 3D neurofibromasphere model. Similar results using cNF-derived SCs were obtained if GPR68 activators were substituted by other cAMP elevators in combination with selumetinib. The promising results obtained in this work open the possibility of using the unbalancing of the Ras/MAPK and cAMP/PKA pathways by a MEKi and a cAMP elevator cotreatment as a therapeutic strategy for cNFs.

## Methods

### Patients, cNFs, and tumor processing.

Patients with NF1 diagnosed according to standard diagnostic criteria (52) provided tumor samples after giving written informed consent. Immediately after excision, tumor samples were placed in DMEM medium (Gibco) containing 10% FBS (Gibco) + 1x GlutaMax (Gibco) + 1x Normocin antibiotic cocktail (InvivoGene) and shipped at room temperature to our laboratories. Tumors were processed as follows: surrounding fat tissue and skin were removed, and tumors were cut into 1 mm pieces and cryopreserved in 10% DMSO (MilliporeSigma) + 90% FBS (Gibco) until used.

### cNF-derived SCs and endoneurial FB cultures.

CNF-derived SCs and endoneurial FBs were isolated as described previously (18). Briefly, cryopreserved cNFs were thawed, cut into smaller pieces using a scalpel, and digested with 160 U/mL collagenase type 1 and 0.8 U/mL dispase (Worthington) for 16 hours at 37ºC, 5% CO_2_.

To establish SC cultures, dissociated cells were seeded onto 0.1 mg/mL Poly-L-lysine– (MilliporeSigma) and 4 μg/mL Laminin-coated (Gibco) dishes in SC media (SCM), which is DMEM (Gibco) with 10% FBS (Gibco), 100 U/mL Penicillin/100 mg/mL Streptomycin (Gibco), 0.5 mM 3-iso-butyl-1-methylxanthine (IBMX, MilliporeSigma), 2.5 μg/mL Insulin (MilliporeSigma), 10 nM Heregulin-b1 (PeproTech), and 0.5 μM Forskolin (MilliporeSigma).

Once the SC culture was established, cells were passaged when they reached confluency with 0.05% Trypsin-EDTA (Gibco) and plated in SCM. Following 24 hours, the culture medium was replaced by SCM without Forskolin, and 24 hours later medium was changed and replaced with SCM for an additional 2–3 days. This process was repeated in cycles. Cells were maintained at 37ºC under a 10% CO_2_ atmosphere. Supplemental Table 3 summarizes all cNF-derived SCs used for each of the distinct functional analyses.

To establish FB cultures, tumor-dissociated cells were plated in DMEM supplemented with 10% FBS media and 1x GlutaMAX (Gibco) and 100 U/mL/Penicillin/100 mg/mL Streptomycin (Gibco). Cells were passaged when necessary with 0.25% Trypsin-EDTA and maintained at 37ºC under a 5% CO_2_ atmosphere.

### SC and endoneurial FB coculture experiment.

70% SC and 30% FB cocultures were grown under SC culture conditions with some modifications: cells were seeded onto 0.1 mg/mL Poly-L-lysine– (MilliporeSigma) and 4 μg/mL Laminin-coated (Gibco) dishes in SCM without IBMX (MilliporeSigma) to favor FB growth. A total of 5 × 10^4^ cells per cm^2^ were seeded, and 24 hours later, the medium was changed to SCM without IBMX or Forskolin. Cocultures were maintained at 37°C under a 10% CO_2_ atmosphere for a total of 72 hours. At this point, supernatants were collected for further analysis, and cells were trypsinized using 0.25% Trypsin-EDTA. A fraction of cells was analyzed for p75 expression by flow cytometry, and another fraction was pelleted and frozen for RNA extraction.

### Neurofibromasphere generation.

Neurofibromaspheres were prepared following the protocol described in Mazuelas et al. (53). In summary, *NF1*^–/–^ iPSC-derived neural crest cells were plated in SC differentiation media for 5 days. At this point, cells were mixed with human cNF-derived FBs and plated in microcavity Aggrewell plates (Stemcell Technologies) to generate neurofibromaspheres. 24 hours after seeding, neurofibromaspheres were treated with vehicle (DMSO), single treatments, or cotreatments for 72 hours.

### DNA extraction.

Genomic DNA from tumors was extracted using the Gentra Puregene Kit (Qiagen) following the manufacturer’s instructions, after homogenization using Tissue Lyser (Qiagen). Genomic DNA from primary cells was extracted using the Maxwell 16 purification Instrument (Promega) following the manufacturer’s instructions.

### RNA extraction.

Tumors were thawed in DMEM supplemented with 10% FBS and homogenized using a TissueRuptor II (Qiagen), and total RNA was extracted using the TriPure Isolation Reagent (Roche) following the manufacturer’s instructions. Total RNA from primary cells was extracted using the 16 LEV simplyRNA Purification Kit (Promega), following the manufacturer’s instructions, in the Maxwell 16 Instrument (Promega). RNA was quantified with a Nanodrop 1000 spectrophotometer (Thermo Fisher Scientific). Quality was assessed with a Bioanalyzer 2200 TapeStation (Agilent).

### NF1 genetic analysis.

*NF1* germline and somatic mutations were detected by *NF1* gDNA sequencing using the I2HCP NGS custom panel (33). Germline mutations were confirmed by DNA Sanger sequencing from cultured cNF-derived FB cells. Loss of heterozygosity of *NF1* locus was detected by Microsatellite Multiplex PCR Analysis of chromosome 17 (54). The reference sequence used was NG_009018_1; NM_000267_3; NP_000258.1. For intragenic deletions we used NM_001042492.2.

### Exome sequencing.

The exome was captured using an Agilent SureSelect Human All Exon V5 kit and sequenced in a HiSeq instrument (Illumina) at Centro Nacional de Analisis Genomicos (Barcelona, Spain). Sequencing reads were then mapped with bwa mem (55) against GRCh38. We called variants with strelka2 (56) and annotated them with annovar (57).

### RNA-Seq analysis.

RNA-Seq libraries were prepared in the IGTP Genomics Core Facility using the TruSeq stranded mRNA Illumina, quantified with the KAPA library quantitation kit for Illumina GA and the Agilent Bioanalyzer, and sequenced at Centro Nacional de Analisis Genomicos in a HiSeq platform pooling 3 samples per lane (paired end, 2 × 100).

To evaluate the effect of the heterotypic interactions on the coculture transcriptomic profiles we created a matched set of virtual cocultures by in silico mixing sequencing reads from the pure SCs and FBs. To build them, we randomly sampled a total of 38.5 million reads from the fastq files of the SCs and FBs in each coculture in the exact same proportion of SCs and FBs determined by flow cytometry analysis. These reads were then stored in a new fastq file representing the expected transcriptional profile of each coculture if no heterotypic interactions were present. The differential expression analysis between each real coculture and its virtual counterpart revealed the transcriptional changes due to heterotypic interactions. RNA-Seq data from real and in silico mixed samples was aligned with Salmon v1.8.0 (58) against the reference genome and transcriptome (refMRNA and hg38 genome from UCSC) (58). We imported transcript-level estimates into R and summarized them to gene level using tximport (59). We then filtered out genes with fewer than 5 counts in more than 2 samples and used DESeq2 (60) to perform differential expression analysis. We finally used clusterProfiler (61) to determine Gene Ontology and KEGG pathway enrichment from differentially expressed genes (adjusted *P* < 0.05). Heatmaps were created using the heatmap R package.

### Flow cytometry of p75 in single cNF-derived cultures and cocultures.

For flow cytometry analysis of p75 in primary SC and FB cultures and SC-FB cocultures, cells were detached with 0.25% Trypsin-EDTA, washed with 1% BSA (MilliporeSigma) in PBS, incubated for 30 minutes on ice with unconjugated primary antibody p75 (1:1,000, Ab3125, Abcam), washed with 1% BSA in PBS, and incubated with Alexa Fluor 488–conjugated secondary antibodies 1:1,000 for 30 minutes on ice. Cells were analyzed by flow cytometry using BD LSR Fortessa SORP and BD FACSDiva 6.2 software.

### Immunofluorescence.

Cells were fixed in 4% paraformaldehyde (Chem Cruz) in PBS for 15 minutes at room temperature, permeabilized with 0.1%Triton-X100 in PBS for 10 minutes, blocked in 10% FBS in PBS for 15 minutes, and incubated with primary antibodies, p75 (1:100, Ab3125, Abcam), S100B (1:1,000, Z0311, Dako), and MPZ (1:100, Ab183868, Abcam), overnight at 4ºC. Secondary antibodies included Alexa Fluor 488 and Alexa Fluor 568 (Thermo Fisher Scientific). Nuclei were stained with DAPI, and images were captured using LEICA DMIL6000 and LAS X Software.

### Measurement of cytokines and chemokines in coculture supernatants.

Supernatants from SC-FB cocultures were collected 72 hours after seeding the cells, transferred into clean polypropylene microcentrifuge tubes, and centrifuged at 16,000 g for 10 minutes at 4ºC to remove any cellular debris. The clarified media were aliquoted into clean polypropylene microcentrifuge tubes and stored at –80ºC until used.

On the day of the analysis, frozen samples were thawed on ice and centrifuged at 16,000 g for 10 minutes at 4ºC. Selected cytokines and chemokines, including BDNF, sICAM-1, and NCAM (catalog HNDG3MAG-36K); S100B and GDNF (catalog HCYTOMAG-60K); and EGF, FGF2, Eotaxin, TGFα, G-CSF, fractalkine, MCP-3, PDGF-AA, IL-1α, IL-1β, IL-6, IL-8, CXCL10, MCP-1, MIP-1α, RANTES, and VEGF (catalog HCYTOMAG-60K), were quantified using Milliplex MAP Luminex microbead assays according to the manufacturer’s instructions (Merck Millipore). Samples were analyzed without dilution in duplicate, and plates were analyzed on a Luminex 200 with xPONENT software (Luminex Corp.).

### Drug treatment.

Drugs used in this study were purchased from commercial sources: selumetinib (Tocris, catalog 6815), ogerin (Tocris, catalog 5722), GANT61 (Tocris, catalog 3191), LGK-974 (Selleckchem, catalog S7143), PAM71 (MedChemExpress, catalog HY-134494), 8-(4-Chlorophenylthio)adenosine 3′,5′-cyclic monophosphate (8CPT cAMP) (Abcam, catalog ab120424), and Forskolin (MilliporeSigma, catalog F6886). They were prepared as indicated by the manufacturers. For drug treatments, cells were seeded onto 0.1 mg/mL Poly-L-lysine– (MilliporeSigma) and 4 μg/mL Laminin-coated (Gibco) plates (SCs and cocultures) or noncoated plates (FBs), and 24 hours later, drugs were added at the indicated concentrations. A vehicle-treated control (DMSO) was applied to each cell type at the same dilution used to deliver the drug.

### Cell viability analysis.

Cell viability in primary SCs, FBs, and cocultures was assessed using the RealTime-Glo MT Cell Viability Assay (Promega). Briefly, cells were seeded onto 0.1 mg/mL Poly-L-lysine (MilliporeSigma) and 4 μg/mL Laminin-coated (Gibco) opaque 96-well plates in SCM (SCs and cocultures) or noncoated opaque 96-well plates in DMEM 10%FBS (FBs) at a density of 500 cells/well. 24 hours later different treatments were added in SCM without IBMX or Forskolin (SCs and cocultures) or DMEM 10%FBS (FBs) together with the RealTime-Glo reagent following the manufacturer’s instructions. Luminescence was monitored for 72 hours on a Varioskan Flash plate reader (Thermo Fisher Scientific). Cells from 3 independent patients were used (see Supplemental Table 3).

Cell viability in neurofibromaspheres was assessed using the CellTiter-Glo 3D luminescence Cell Viability Assay (Promega). Briefly, neurofibromaspheres were generated in 96-well spheroid microplates (Corning) at a density of 5,000 cells per spheroid/well (3,500 iPSC-derived differentiating SCs and 1,500 cNF-derived FBs) in a total volume of 200 μL. 24 hours later, 100 μL of cell culture was removed, and drugs were added at twice their previous concentrations. Cell viability was measured after 72 hours of drug exposure following the manufacturer’s instructions on a GloMAX Explorer plate reader (Promega).

### Cell proliferation analysis.

Cell proliferation was assessed using the Click-iT EdU Flow Cytometry Assay Kit (Thermo Fisher Scientific). One hundred thousand primary SCs from 3 independent patients (see Supplemental Table 3) were plated onto 0.1 mg/mL Poly-L-lysine (MilliporeSigma) and 4 μg/mL Laminin-coated (Gibco) 12-well plates in SCM without IBMX. 24 hours later drugs were added in SCM without IBMX or Forskolin. 48 hours after drug treatment cells were treated with 5 μM EdU for 16 hours, fixed, permeabilized, and click labeled with Alexa Fluor 488 azide according to the manufacturer’s protocol (Thermo Fisher Scientific). Cells were also stained with propidium iodide to detect DNA content. Data were collected and analyzed using an FACSCanto II (BD Biosciences) and BD FACSDiva 6.2 software.

### Apoptosis analysis.

One hundred thousand primary SCs from 3 independent patients (see Supplemental Table 3) were plated onto 0.1 mg/mL Poly-L-lysine (MilliporeSigma) and 4 μg/mL Laminin-coated (Gibco) 12-well plates. 24 hours later drugs were added in SCM without IBMX or Forskolin. 48 hours after drug treatment, cells were harvested using trypsin-EDTA 0,05%, washed in PBS, and incubated with Annexin-V-FITC antibody using the Annexin V FITC Apoptosis detection kit (Invitrogen) following the manufacturer’s instructions. Data were collected and analyzed using a FACSCanto II (BD Biosciences) and BD FACSDiva 6.2 software.

### Quantification of cAMP levels.

Intracellular cAMP levels were quantified using the cAMP-Glo Max Assay (Promega) following the manufacturer’s instructions. Five thousand cNF-derived primary SCs from 3 independent patients (see Supplemental Table 3) were plated onto 0.1 mg/mL Poly-L-lysine (MilliporeSigma) and 4 μg/mL Laminin-coated (Gibco) opaque 96-well plates in SCM without IBMX with Forskolin and maintained at 37ºC under a 10% CO_2_ atmosphere for 24 hours. At this point, cells were treated with an induction buffer containing ogerin, Forskolin (as a positive control), or neither of them for 30 minutes at room temperature, and luminescence was monitored on a Varioskan Flash plate reader (Thermo Fisher Scientific). The induction buffer was composed of PBS without calcium or magnesium, 500 μM IBMX, 100 μM Ro 20-1724 (MilliporeSigma, B8279), and 25 mM MgCl_2_.

### Recovery experiment.

Thirty thousand primary SCs from 3 independent patients (see Supplemental Table 2) were plated onto 0.1 mg/mL Poly-L-lysine (MilliporeSigma) and 4 μg/mL Laminin-coated (Gibco) 8-well chamber slides in duplicate. 24 hours later drugs were added in SCM without IBMX, and 3 days later medium was replaced with fresh drugs. After 6 days of treatment 1 chamber slide was fixed in 4% PFA, and the other one was maintained in culture for 3 more days with SCM without drugs and then fixed with PFA 4%. Fixed cells were stained with MPZ antibody and nuclei were stained with DAPI. To quantify cell recovery, nuclei from 5 representative ×20 fields were counted per time point and treatment.

### Statistics.

Data were analyzed and graphically represented using Microsoft Office Excel spreadsheets and GraphPad Prism (version 9.4.1). Quantitative data are shown as the mean ± SEM of 3 independent experiments.

Bioinformatic analysis of RNA-Seq is described in *RNA-Seq analysis*, including the software and statistical methods used. We applied the default multiple testing correction recommended by the different software packages when applicable. The significance level was established at *P* < 0.05 unless otherwise stated.

Statistical analysis of apoptosis data was conducted using a 1-tailed unpaired *t* test (*P* ≤ 0.05).

For neurofibromasphere analysis, disaggregation index and raw integrity density (RawIntDen) from each fluorescent channel were calculated using ImageJ (NIH) software. Disaggregation index was calculated by measuring disaggregated spheroid area normalized by spheroid area at 72 hours of treatment and cotreatment. To identify the RawIntDen for each channel we used the filter Gaussian blur and threshold functions to better detect structures. Once structures were selected we measured RawIntDen from each channel (live and death) at 72 hours of treatment and cotreatment. Data are shown as the median of *n* ≥ 3 spheroids. One-tailed unpaired *t* test (*P* ≤ 0.05) with a Holm-Bonferroni post hoc adjustment was performed.

For the recovery experiment, a 1-tailed unpaired *t* test (*P* ≤ 0.05) was performed between selumetinib treatment and selumetinib-ogerin cotreatment.

The meaning of the value of *n*, and/or dispersion and precision measure (SEM), can be found in the figure legends and Results section.

### Study approval.

This study was reviewed and approved by the IGTP Institutional Review Board. Written informed consent was received from all patients with NF1 that provided tumor samples prior to participation in this study.

### Data availability.

All data needed to evaluate the conclusions are present in the manuscript or the Supporting Data Values file. RNA-Seq data have been deposited at the NF Data Portal in Synapse (https://doi.org/10.7303/syn11374353) and are available under a data access agreement. Any additional information required to analyzed the data reported in this paper are available from ES, MC, or BG upon request.

## Author contributions

HM, MC, BG, and ES designed the research studies. HM, IUA, and MC conducted the experiments. MML and BG performed bioinformatic design and analysis. HM, AN, IR, IB, EC, and CL acquired and analyzed genetic data. CL provided reagents. HM, MML, MC, BG, and ES generated the figures. HM, MC, and ES wrote the manuscript. HM, MML, IUA, AN, IR, IB, EC, CL, MC, BG, and ES reviewed and edited the manuscript.

## Supplementary Material

Supplemental data

Supplemental data set 1

Supplemental data set 2

Supplemental data set 3

Supporting data values

## Version 1 01/04/2024

In-Press Preview

## Version 2 02/08/2024

Electronic publication
