# Supplementary material for: Unbalancing cAMP and Ras/MAPK pathways as a therapeutic strategy for cutaneous neurofibromas
Source: JCI Insight. 2024 Jan 4;9(3):e168826. doi: 10.1172/jci.insight.168826 (PMC11143965; doi:10.1172/jci.insight.168826)
Supplement: Supplemental data [file jciinsight-9-168826-s028.pdf]

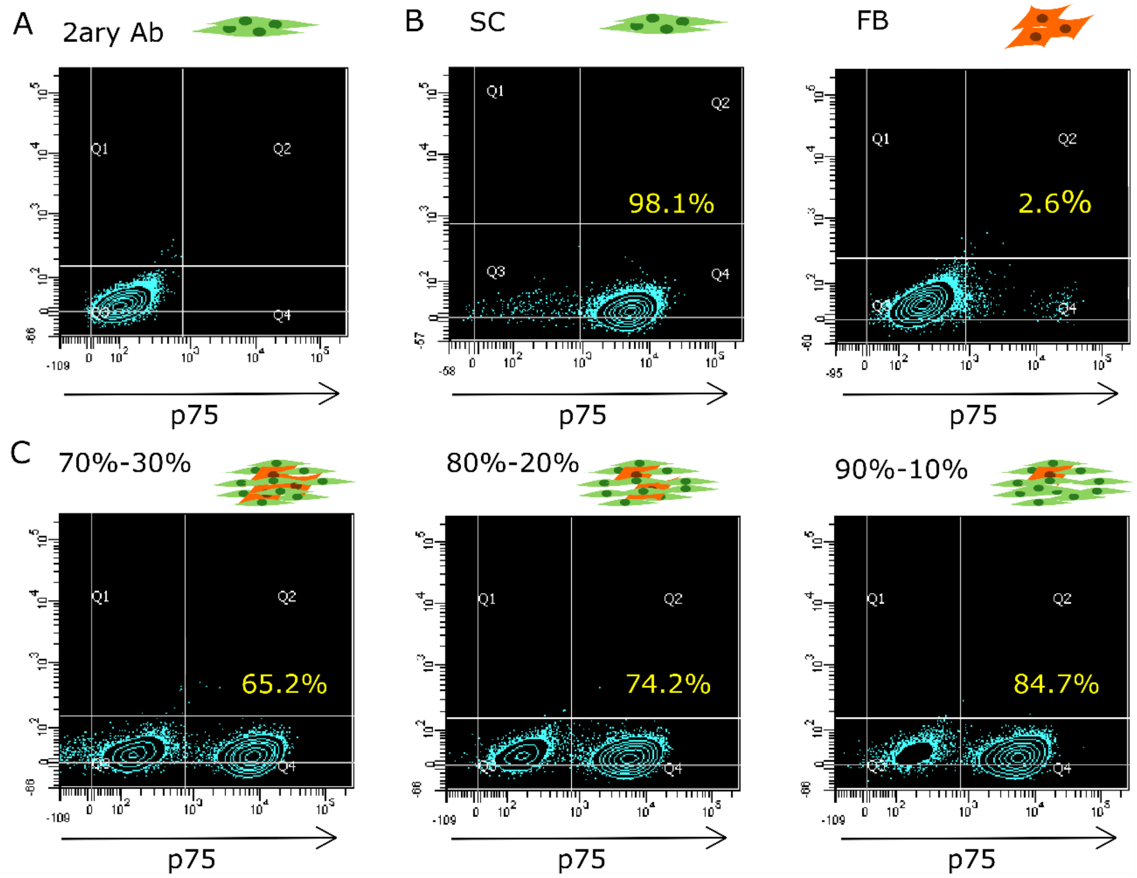

Supplemental Figure 1. Setting up SC-FB co-culture conditions. Seeding 70% SC– 30% FB co-culture is enough to obtain a percentage of cells at 72 hours, closely resembling cNF composition. Flow cytometry analysis of p75 in single SC and FB cultures and different proportion of SCs and FBs co-cultures.

A) Control SCs incubated with the secondary antibody. **B)** Single SC and FB cultures incubated with p75 primary antibody and the secondary antibody. **C)** Percentages of p75 positive cells in SC-FB co-cultures after 72h, at different initial seeding conditions (70% SCs– 30% FB; the 80% SCs– 20% FB and the 90% SCs – 10% FB; respectively). Percentages of p75 positive cells are indicated in yellow. SC: Schwann cell; FB: Fibroblast. **Related to Figure 1**.

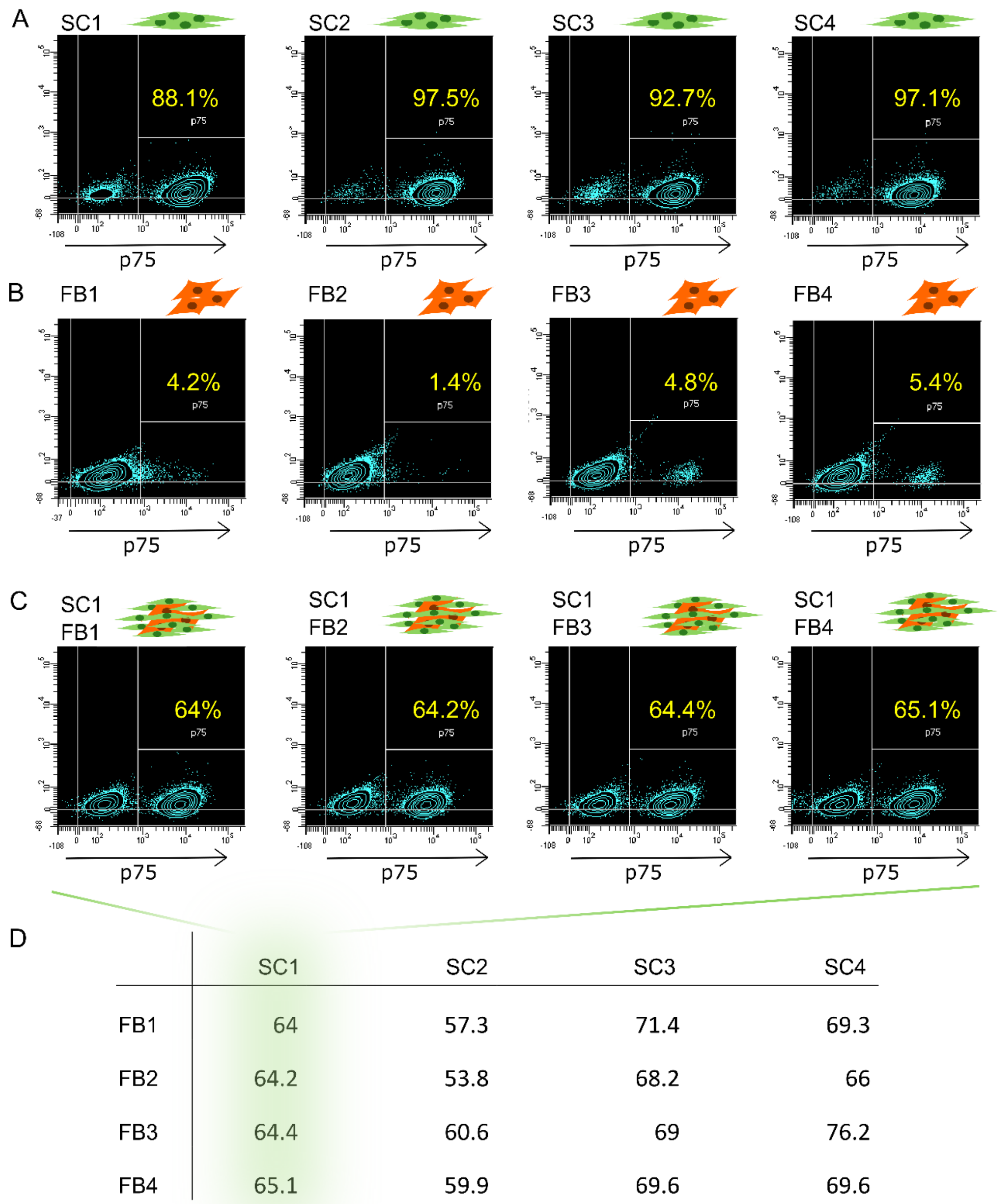

D

	SC1	SC2	SC3	SC4
FB1	64	57.3	71.4	69.3
FB2	64.2	53.8	68.2	66
FB3	64.4	60.6	69	76.2
FB4	65.1	59.9	69.6	69.6

Supplemental Figure 2. p75 expression in co-cultures as a control of co-culture composition for RNA-seq analysis. Flow cytometry analysis for p75 of single SC and FB cultures and SC-FB co-cultures of four single cNF-derived SC cultures, four single cNF-derived FB cultures, and four different co-culture combinations at 72 hours are shown. The exact percentage of cells expressing p75 is shown in yellow **A)** Four independent single SC cultures. **B)** Four independent single FB cultures. **C)** Four independent co-cultures combinations using SC1 Schwann cells and FB from the four independent cNFs (FB1, FB2, FB3, and FB4). **D)** Summary of the different percentages of p75-expressing cells in the sixteen different co-culture combinations at 72 hours. SC: Schwann Cells; FB: Fibroblasts. **Related to Figure 1 and 2**.

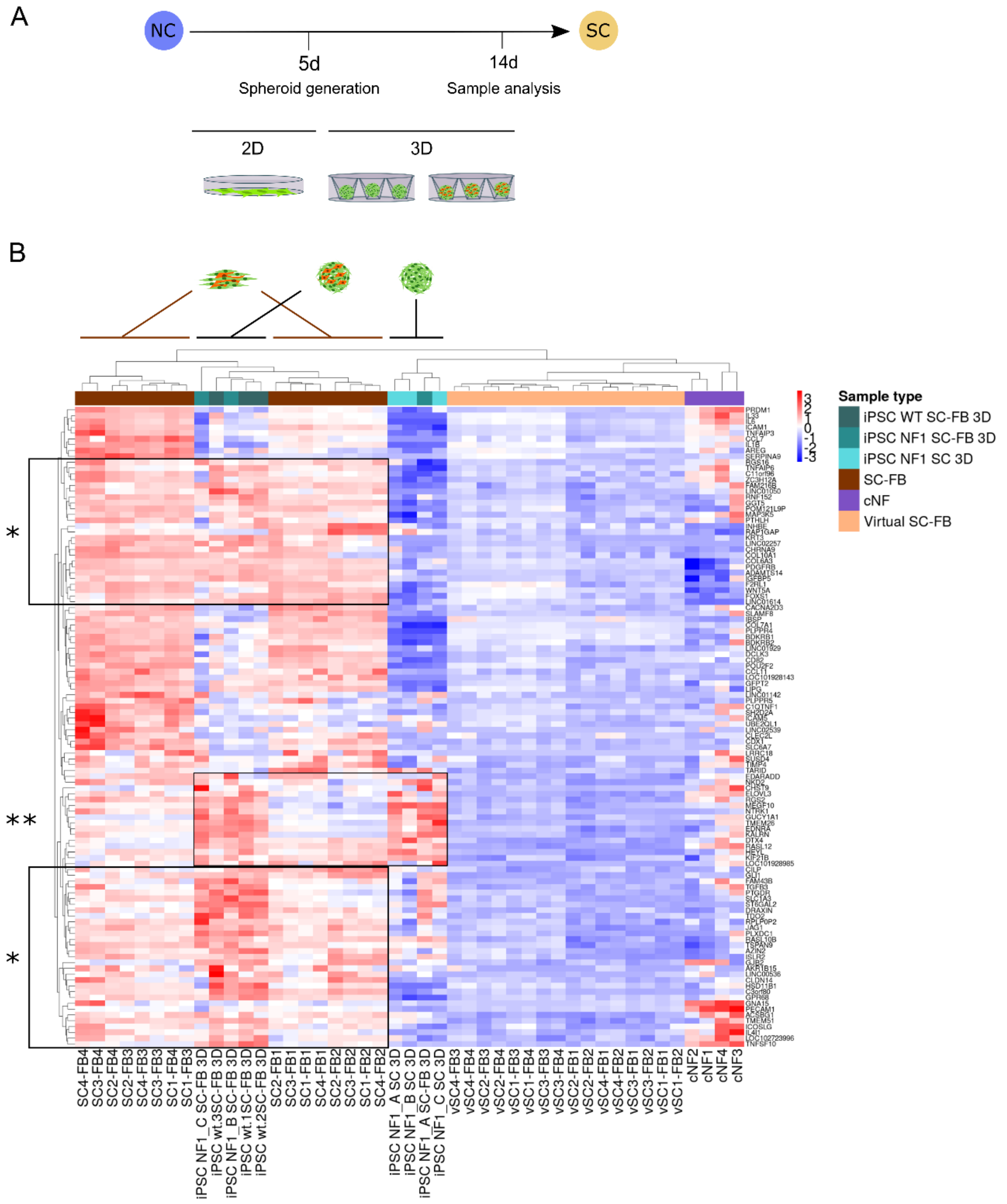

Supplemental Figure 3. Validation of the SC-FB crosstalk signature in an iPSC-derived neurofibromasphere model. iPSC-derived SC-FB 3D co-cultures express most genes upregulated in cNF-derived SC-FB co-cultures. **A)** Schematic representation of the iPSC-based SC or SC-FB model in 3D from Mazuelas et al. 2022. **B)** Heatmap showing the unsupervised cluster analysis of differentially upregulated genes in SC-FB co-cultures for different samples: iPSC-derived *NF1*(-/-) SC 3D culture (light turquoise); iPSC-derived SC-FB 3D co-culture (dark turquoise) spheroids generated from *NF1*(+/+) (WT, dark) and *NF1*(-/-) (NF1) iPSCs; real (brown) and virtual (salmon) SC-FB co-cultures; and in cNFs (purple). The expression color ranges from dark blue, showing down-regulated genes, to red, showing up-regulated genes. WT: wild type, SC: Schwann Cell, FB: Fibroblast; NC: Nerual Crest. * expression profile that occurs due to SC-FB interaction, wether in 2D o 3D. ; ** expression profile that occurs due to SC-SC interaction. **Related to Figure 2**.

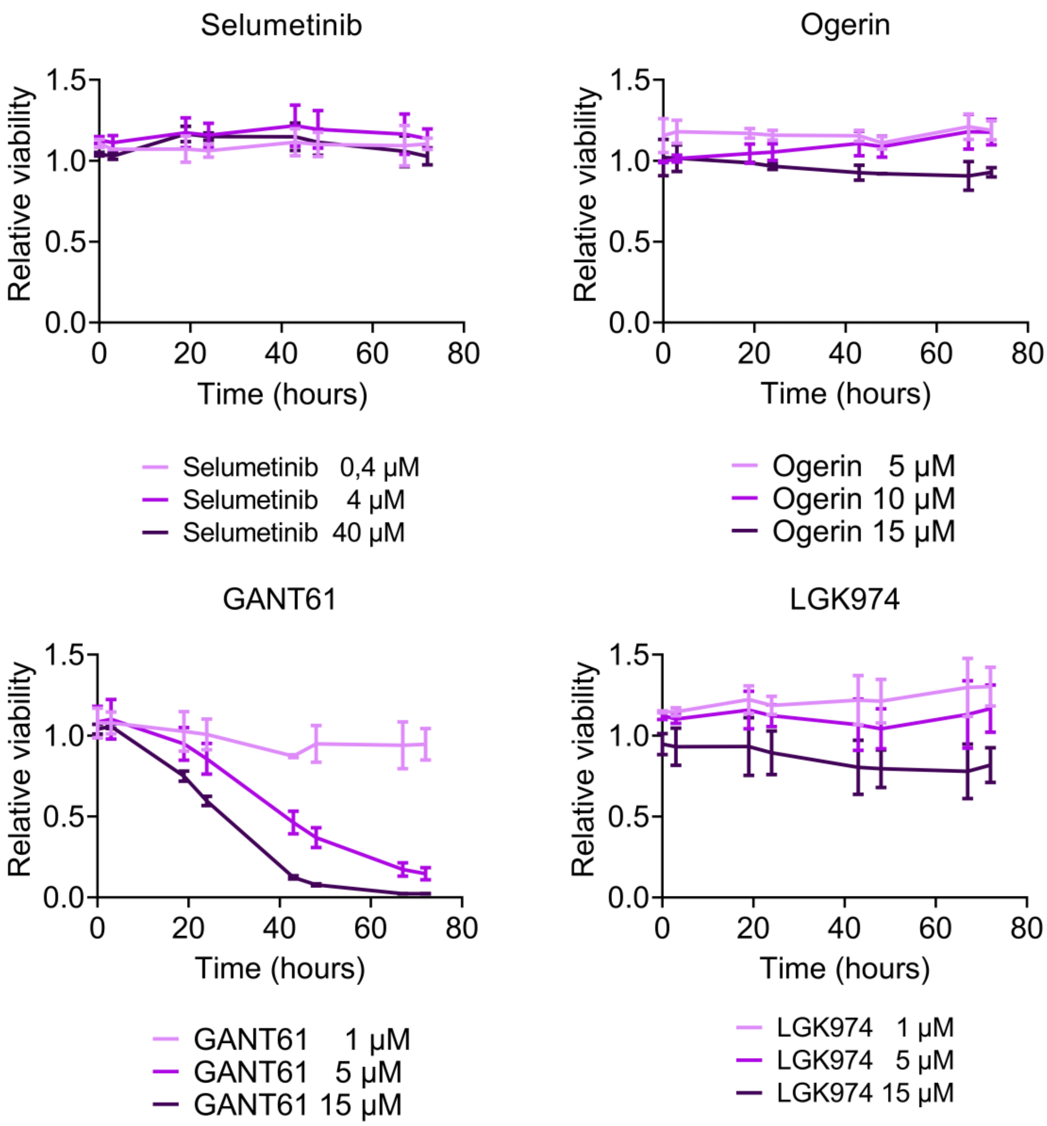

Supplemental Figure 4. Toxicity effect of single drugs tested on NF1 patient skin fibroblasts.

NF1 patient skin fibroblasts were plated and 24h later treated with different doses of Selumetinib, Ogerin, GANT61, and LGK974. Cell viability was monitored throughout 72h using RealTime-Glo MT Cell Viability Assay. Data are expressed as mean +/- SEM from three different fibroblast cultures, related to DMSO-treated control cells. **Related to Figure 5.**

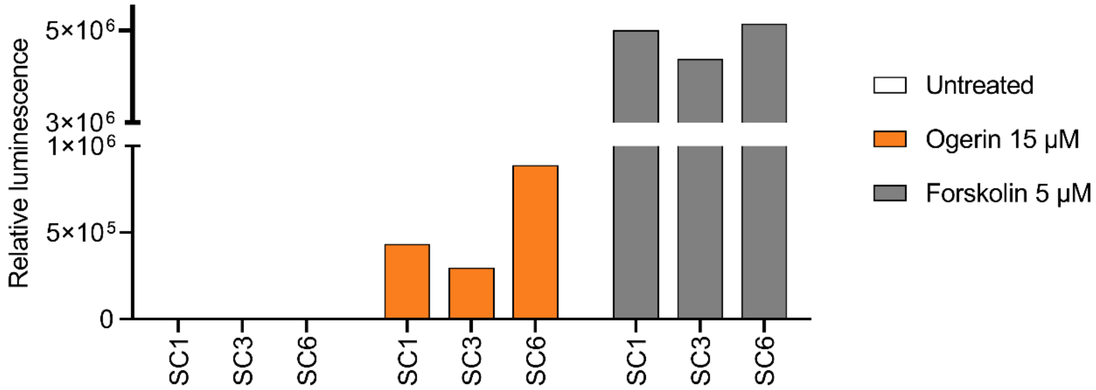

Supplemental Figure 5. Ogerin treatment increases cAMP levels in cNF-derived SCs. Intracellular cAMP levels were quantified using the cAMP-Glo Max Assay (Promega). SCs were plated in SCM without IBMX with Forskolin and maintained at 37ºC under a 10% CO2 atmosphere for 24 hours. At this point, cells were treated with induction buffer containing Ogerin or Forskolin or none for 30 minutes at room temperature. cAMP-Glo Max Assay was performed following manufacturer's instructions, and Luminescence was monitored on a Varioskan Flash plate reader (Thermo). Relative luminescence represents the values from treated compared to untreated. **Related to Figures 5 and 6.**

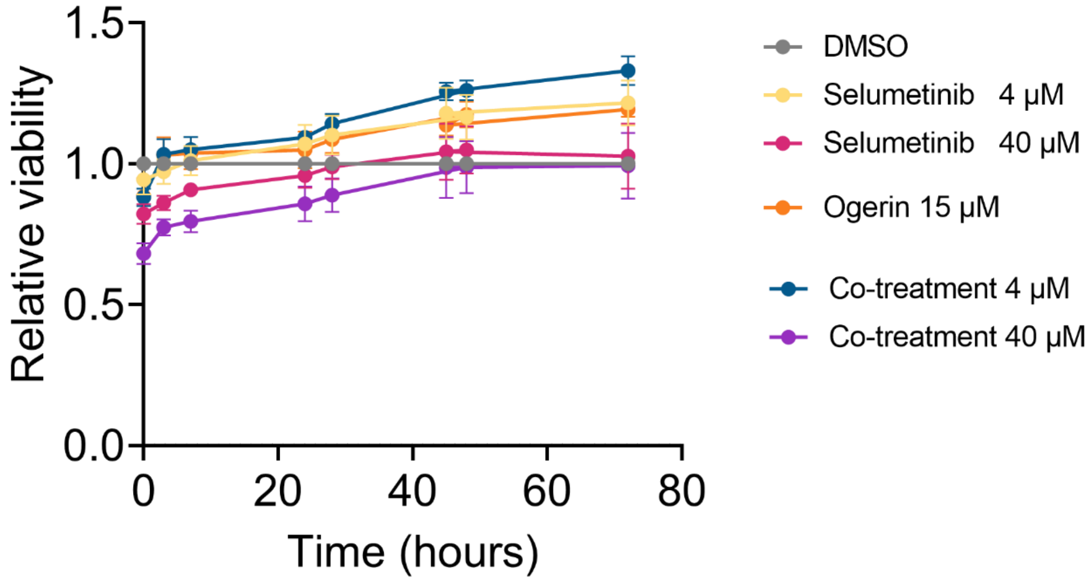

Supplemental Figure 6. Toxicity effect of Selumetinib-Ogerin co-treatment in NF1 skin fibroblasts. Cells were plated and 24h later treated with single drugs (4 µM Selumetinib, 40 µM Selumetinib, 15 µM Ogerin) or combination of drugs (4 µM Selumetinib and 15 µM Ogerin; 40 µM Selumetinib and 15 µM Ogerin). Cell viability was monitored throughout 72h using RealTime-Glo MT Cell Viability Assay. Data are expressed as mean +/- SEM from three different fibroblast cultures, related to DMSO-treated control cells. **Related to Figure 6.**

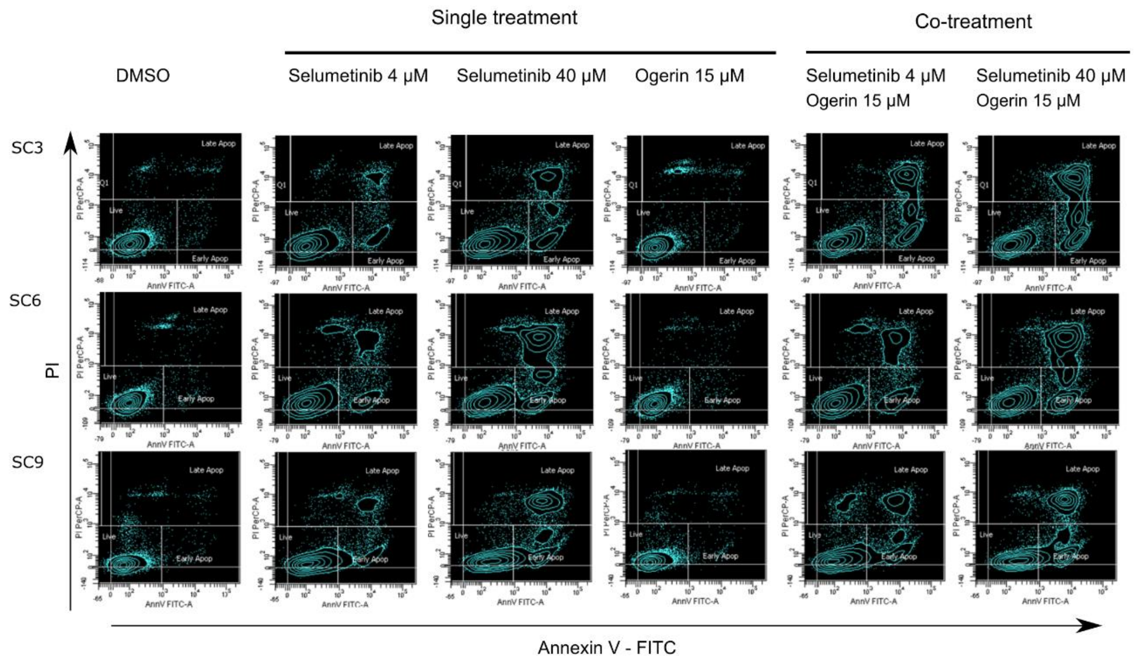

Supplemental Figure 7. Effect of Selumetinib and Ogerin treatments and co-treatments on cell death in primary SC cultures. Cytometry plots showing Annexin V and Propidium Iodide (PI) staining after 48h of treatments. Cells were plated and 24h later treated with vehicle (DMSO), single drugs (4 µM Selumetinib, 40 µM Selumetinib, 15 µM Ogerin) or co-treatment of drugs (4 µM Selumetinib and 15 µM Ogerin; 40 µM Selumetinib and 15 µM Ogerin) for 48 h. At this point, apoptosis was quantified by flow citometry using the Annexin V FITC Apoptosis detection kit (Invitrogen). **Related to Figure 6.**

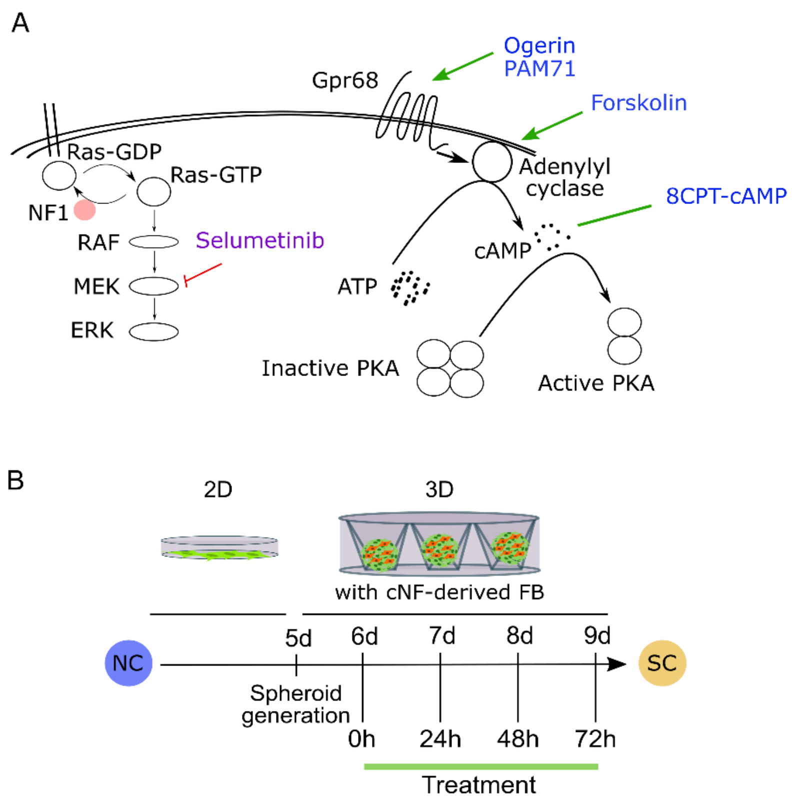

Supplemental Figure 8. Schematic representations. A) Ras/MAPK and cAMP/PKA signaling pathways and the different compounds used to inhibit or activate different elements. **B)** Schematic representation of drug treatment in iPSC-based SC-FB cNF model in 3D (neurofibromaspheres). *NF1*(-/-) iPSC-derived NC cells were plated in SC differentiation media for 5 days. At this point, cells were mixed with cNF-derived FB and plated in microcavity Aggrewell plates to generate neurofibromaspheres. 24h after seeding, neurofibromaspheres were treated with single treatments and co-treatments for 72h.

Supplemental Table 1. **Summary of constitutional and somatic *NF1* alterations in selected human cNFs from which SC-FB co-cultures were established**

Patient information			*Tumor information*		
Patient ID	Sex	*NF1* constitutional mutation	Tumor ID	Age at resection	*NF1* somatic mutation
1	M	c.5898_5899delGA	cNF1_1	46	c.4120C>T
2	M	c.3826C>T	cNF1_2	34	c.4537C>T
3	F	c.4309G>T	cNF1_3	34	c.810_833delAATCATTCTCCTTATCTTGTGTCC
4	F	c.3233C>G	cNF1_4	76	c.3158C>G

Supplemental Table 2. List of candidate genes and activators and inhibitors used.

Candidate	Signaling pathway	Inhibitors/ Activators		
		Type	Name	Reference
AREG	EGF<br>TGFalpha	Neutralizing Antibody	Human Amphiregulin mAb (Clone 31221)	R&D<br>MAB262-SP
TGFb3	TGFb	Neutralizing Antibody	TGFb3 mAb (Clone 20724)	R&D<br>MAB243-SP
JAG1	Notch	Neutralizing Antibody	Human Jagged 1 polyclona Ab	R&D<br>AF1277-SP
GLI1	Sonic Hedgehog	Inhibitor	GANT61	TOCRIS<br>3191
WNT5A	Wnt	Inhibitor	LGK-974	Selleckchem S7143
GPR68	AMPc	Activator	Ogerin	TOCRIS<br>5722
TGFA	TGFalpha	Activator	Recombinant Human TGF alpha	Abcam<br>ab233681
Positive control	ERK	Inhibitor	Selumetinib	TOCRIS<br>6815

Supplemental Table 3. Summary of human cNFs used in functional assays using single cultures of SCs, FBs and SC-FB co-cultures.

			Real Time Cell Titer-Glo Viability Assay			Proliferation Assay (EdU)	Real Time Cell Titer-Glo Viability Assay	Apoptosis Assay	cAMP-Glo Max Assay	Real Time Cell Titer-Glo Viability Assay	Real Time Cell Titer-Glo Viability Assay	SC recovery experiment
NF1 patient ID	Sex	SC-Fb co-culture<br>Fig2	SC<br>Fig5C	FB<br>Fig5C	SC-FB co-culture<br>Fig5C	Fig5D	SC Selumetinib-Ogerin Co-treatment<br>Fig6A	SC Co-treatment<br>Fig6B	SC Ogerin treatment<br>FigS5	SC Selumetinib-Ogerin/ PAM71 co-treatment<br>Fig6D	SC Selumetinib-8CPT cAMP/ Forskolin co-treatment<br>Fig6E	Fig8C
1	M	X	X	X	X				X	X		
2	M	X										
3	F	X	X	X	X	X	X	X	X	X	X	X
4	F	X				X					X	X
5	F						X					
6	M			X				X	X			
7	M						X					
8	M		X		X	X				X	X	
9	F							x				X
